# Neuromorphic Photoresponse in Ultrathin SnS_2_‑Based Field Effect Transistor

**DOI:** 10.1021/acsami.5c11651

**Published:** 2025-08-26

**Authors:** Sebastiano De Stefano, Ofelia Durante, Andrea Sessa, Antonio Politano, Gianluca D’Olimpio, Tsotne Dadiani, Enver Faella, Adrian Dinescu, Catalin Parvulescu, Crispin Hetherington, Chia-Nung Kuo, Chin Shan Lue, Martino Aldrigo, Maurizio Passacantando, Antonio Di Bartolomeo

**Affiliations:** † Department of Physics “E. R. Caianiello”, University of Salerno, Fisciano (SA), 84084, Italy; ‡ 201850 University of L‘Aquila, Department of Physical and Chemical Sciences, L’Aquila, 67100, Italy; § 172906 National Institute for Research and Development in Microtechnologies, Voluntari (Ilfov), 077190, Romania; ∥ National Center for High Resolution Electron Microscopy, Centre for Analysis and Synthesis, Lund University, Lund, SE-22100, Sweden; ⊥ Department of Physics, 34912 National Cheng Kung University, Tainan, 70101, Taiwan; ○ Taiwan Consortium of Emergent Crystalline Materials (TCECM), National Science and Technology Council, Taipei 10601, Taiwan

**Keywords:** 2D materials, field-effect transistors, photoresponse, trap states, neuromorphic

## Abstract

As artificial intelligence continues to evolve, neuromorphic technologies, which emulate biological neural networks, are increasingly seen as a promising direction. Two-dimensional materials are considered promising for neuromorphic applications due to their tunable electrical and optoelectronic properties. In this work, a back-gated tin disulfide (SnS_2_) field-effect transistor (FET) is electrically and optoelectronically characterized at different temperatures (80, 295, and 380 K), pressures (ambient and 10^–4^ mbar), and illumination conditions (dark and laser light from 420 to 800 nm). Responsivity peaks of up to ∼100 A/W are recorded. Persistent photoconductivity is observed, with current retention after illumination ranging from 0% to ∼30% of the initial dark current, depending on temperature and gate voltage. The underlying microscopic mechanisms are analyzed, revealing a key role for trap states and ambient adsorbates, and a qualitative model is proposed to explain the observed effects. Trap states within the bandgap, often considered detrimental, are exploited to induce synaptic plasticity, with synaptic weight changes tunable from 0.001 to 3000. Temperature and gate voltage are found to be effective parameters for modulating plasticity, enabling smooth transitions between short-term and long-term behavior. These results clarify the microscopic origin of plasticity in SnS_2_, demonstrate its robustness under realistic conditions, and lay the foundation for the integration of this two-dimensional material into next-generation neuromorphic architectures.

## Graphical abstract

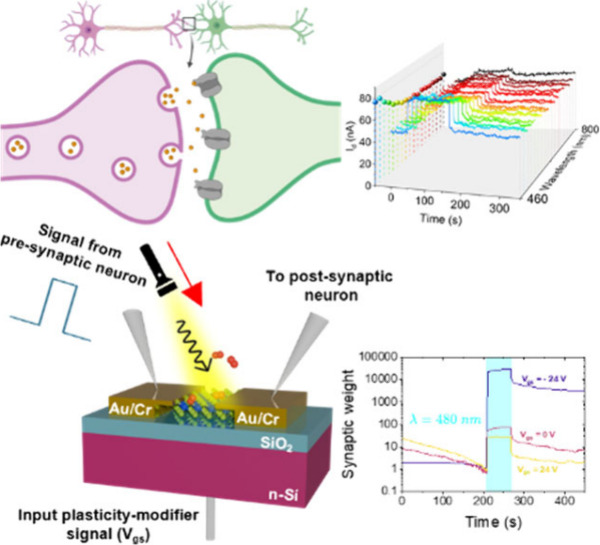

## Introduction

Since the discovery of graphene in 2004, two-dimensional (2D) materials have become a central focus in condensed matter physics, nanoelectronics, and optoelectronics.^1–3^ Their atomically thin structure helps overcome key limitations of silicon-based technologies, particularly short-channel effects in field-effect transistors (FETs), enabling next-generation devices.^4–6^ Among 2D materials, transition metal dichalcogenides (TMDs) such as MoS_2_ and WS_2_ have attracted wide interest due to their layer-tunable electronic properties and bandgaps, and compatibility with flexible, low-power systems and sensors.^7–9^ Beyond traditional TMDs, post-transition metal dichalcogenides are emerging as promising alternatives. Notably, tin disulfide (SnS_2_), a group-IVA compound, combines favorable features: earth abundance, moderate bandgap, and a layered structure with weak interlayer van der Waals forces, allowing easy exfoliation into nanosheets. SnS_2_, like many other 2D materials, exists in numerous crystalline phases that differ from one another in the way the monolayers stack together, which can be selectively obtained depending on the fabrication techniques and conditions employed. For SnS_2_, more than 70 polymorphs have been identified, including 4H, 18R:^10^ the most common structure is the so-called CdI_2_-type (space group *P* 3 *m*1), typically referred to in the 2D materials literature as the 1T structure. The lattice parameters are estimated as a = 3.68 Å for the monolayer, and a = 3.64 Å and c = 5.88 Å for the bulk material.^11^ SnS_2_ offers high charge carrier mobility (∼230 cm^2^ V^–1^ s^–1^) and strong optical absorption (>10^4^ cm^–1^), making it suitable for FETs, photodetectors, and optoelectronic components. SnS_2_-based FETs have achieved on/off ratios up to 10^8^ and photoresponse times as low as 5 μs.^12,13^ In order to improve device performance, strategies like heterostructure engineering, plasmonic enhancement, and quantum dot coupling have been implemented in SnS_2_-based systems.^14,15^ Moreover, as research moves toward neuromorphic computing and systems designed to emulate biological synapses and neural behavior, 2D materials offer critical advantages such as scalability, low energy consumption, and multifunctionality.^16,17^ Several successful emulation attempts have been made, and SnS_2_ is no exception. Recent advances in SnS_2_-based neuromorphic devices have demonstrated the versatility of this layered semiconductor for emulating synaptic functionalities through a variety of mechanisms. Spin-coated SnS_2_ memristors doped with Ca^2+^ have successfully reproduced key synaptic behaviors such as short- and long-term plasticity (STP/LTP), paired-pulse facilitation (PPF), and spike-timing-dependent plasticity (STDP), enabled by ionic migration within the film structure.^18^ In electrochemical synapses based on SnS_2_-reduced graphene oxide composites, Na^+^ intercalation allows for linear and symmetric weight updates with low variability, supporting dynamic spatiotemporal signal processing.^19^ Heterostructures combining SnS_2_ with SnO_2_ or SnO_*x*_ have shown improved resistive switching performance, fast switching times, high on/off ratios, and analog conductance modulation, further validating the potential of defect and interface engineering in neuromorphic systems.^20^ Additionally, floating-gate transistors incorporating SnS_2_ channels have achieved high endurance and retention with excellent linearity in potentiation and depression, and demonstrated their functionality in image recognition tasks.^21^ Here, we present a full electrical and optoelectronic characterization of a back-gated SnS_2_ FET at different temperatures (80, 295, and 380 K), pressures (ambient and 10^–4^ mbar), and illumination conditions (dark and laser wavelengths from 420 to 800 nm). We analyze photoconduction mechanisms, spectral response, and the role of the back-gate and temperature in modulating these effects. Particular attention is given to trap states within the bandgap, likely enhanced by inhomogeneous formation of SnO_2_, which, though typically seen as detrimental, are exploited here to mimic synaptic behavior. These states are key to enabling artificial synaptic functions, positioning SnS_2_ transistors as a viable route toward neuromorphic devices that emulate the dynamics of biological neural systems. The present work introduces a novel approach where synaptic plasticity is induced by optical pulses and deterministically modulated through the gate voltage, enabling the transition between STP and LTP with a single light stimulus. This control strategy, combined with a simple and reproducible device architecture, expands the capabilities of SnS_2_-based neuromorphic platforms by introducing a new degree of freedom in plasticity modulation.

## Material and Device

Figure 1(a) shows the crystalline structure of monolayer 1T-SnS_2_, the polymorph of SnS_2_ used in this study, viewed along the *a*-axis (top) and the *c*-axis (bottom). According to Density Functional Theory (DFT) calculations available in the literature,^11^ the band structure of bulk 1T-SnS_2_ exhibits a valence band maximum along the Γ–M direction and a conduction band minimum located at the L point of the first Brillouin zone, making it an indirect semiconductor with 2.18 eV bandgap. On the other hand, a direct transition along the L→L direction is reported at an energy of 2.61 eV. However, unlike other two-dimensional dichalcogenides such as 2H-MoS_2_,^22^ 1T-SnS_2_ does not undergo a transition from an indirect to a direct bandgap as the number of layers decreases, remaining indirect even in its monolayer form. For 1T-SnS_2_, the valence band maximum remains along the Γ–M line, while the conduction band minimum shifts toward the high-symmetry M point. The associated indirect bandgap for the monolayer is estimated to be 2.41 eV, while the direct bandgap, associated with the M→M transition, is estimated at 2.68 eV.

**Figure 1:**
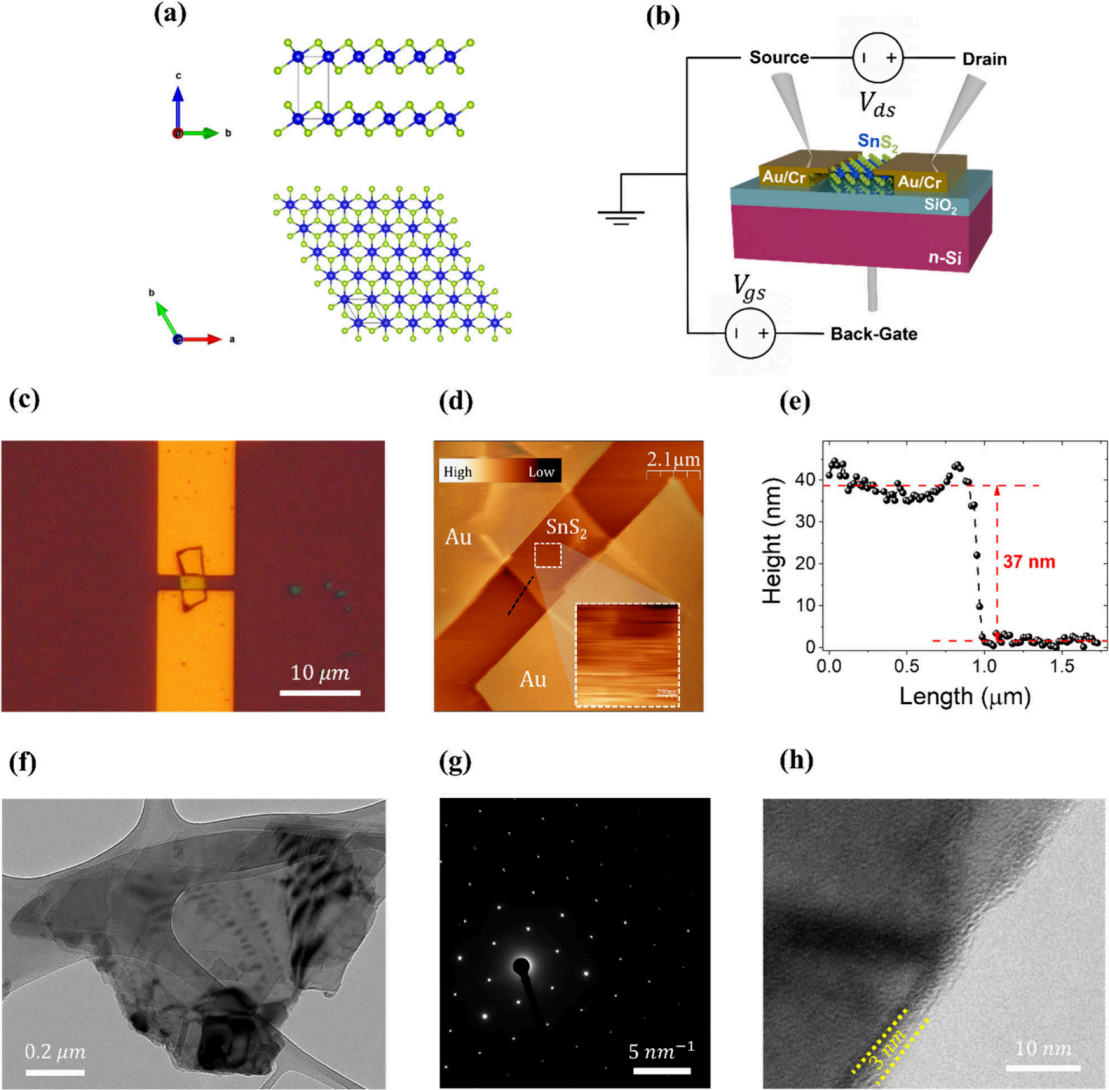
(a) *a*-axis (top) and *c*-axis (bottom) view of 1T-SnS_2_ crystalline structure. (b) Cartoon of the fabricated device with sketched experimental apparatus. (c) Device optical image at 100× magnification (10 μm scale). (d) Device AFM on a scan area of 10.5 μm × 10.5 μm. The color scale ranges from 0 (black) to 500 nm (white) Inset: flake 1 μm × 1 μm scan area to measure the RMS roughness. The color scale for the inset ranges from 0 to 50 nm. (e) Flake height profile acquired from dashed black line in (d). (f) A thin flake imaged in bright field on a 200 kV TEM. (g) A selected area diffraction pattern from the flake at the 0 0 0 1 zone axis and (h) a higher magnification image showing possible small regions of another structure at small sections of the edge.

Multilayer (ML) SnS_2_ flakes were obtained via mechanical exfoliation from a bulk crystal and subsequently transferred onto a SiO_2_/Si substrate with a 290 nm thick SiO_2_ layer. Then, the position (in relation to the alignment marks) and the dimensions of the flakes were measured by optical microscopy in order to define the contact pads. The latter were exposed in 250 nm thick poly­(methyl methacrylate) (PMMA) layer by electron beam lithography (EBL) at 10 kV accelerating voltage and 130 pA beam current. For development, the substrate was immersed in a mixture of methyl isobutyl ketone (MIBK):isopropyl alcohol (IPA) (1:3) for 100 s at room temperature. Then, the substrate was covered with a 20 nm chromium (Cr) adhesion layer followed by a 100 nm gold (Au) layer by e-beam evaporation in a highly directional deposition equipment (TEMESCAL: FC-2000). Finally, for the lift-off step, the sample was placed in acetone at room temperature for 2 h.

Figure 1(b) shows the layout of the device, along with a schematic of the measurement setup. Two probes were used to contact the device pads, while a back-gate contact was fabricated on the scratched n-silicon substrate using silver paste. Figure 1(c) shows an optical image of the device (100× magnification) in which a flake, approximately rectangular in shape and partially covered by the contacts, is distinguishable. The channel dimensions of length L = 1.86 μm and width W = 3.21 μm result in a total effective area A = 5.97 μm^2^. Figure 1(d) shows an Atomic Force Microscope (AFM) image of the device, and the scan area is 10.5 μm × 10.5 μm. The Root Mean Square (RMS) roughness, acquired on an area of 1 μm × 1 μm of flake (see dashed white square in AFM image and inset), is 8 nm. As pristine monolayer or few-layer SnS_2_ has a roughness on the order of 0.2–0.5 nm. The height profile in Figure 1(e), acquired along the dashed black line of Figure 1(d), indicates an average thickness of about 37 nm, corresponding approximately to a 60-layer thick flake.^23^

Figure 1(f) shows a Transmission Electron Microscope (TEM) image of a flake with length and width of the order of 1 μm, and it is clearly thin. An electron diffraction pattern from the flake is shown in Figure 1(g). The hexagonal nature of the structure is apparent. Higher resolution images such as the one shown Figure 1(h) indicated that the flakes were not coated by an oxide. Some regions however showed sections of amorphous at the edges. Elemental maps (not shown here) confirmed the presence of Sn and S across the flakes.

## Results and Discussion

### Electrical Characterization

The device was initially placed at a base pressure (P) equal to 10^–4^ mbar. Electrical characterization was carried out by measuring the drain current (I_d_) versus the drain voltage (V_ds_), hereafter referred to as the IV characteristic, at different temperatures (T), with the gate contact grounded and under dark conditions. As shown in Figure 2(a) (and in Figure S1 on linear scale), the IV curves, acquired with V_ds_ swept from −5 to 5 V, at T = 80, 295, and 380 K, exhibit a nearly symmetric behavior. This suggests symmetric contacts and the absence of a significant Schottky barrier at the Au/Cr–SnS_2_ interface.^24^ The temperature dependence of the device resistance reported in Figure 2(b), estimated through the IV linear fit, does not follow a monotonic trend. A similar behavior has been previously observed in similar devices fabricated with the same material.^12^ Similarly, the I_d_-V_gs_ curves, referred to as the transfer characteristics, were acquired by sweeping the back-gate voltage (V_gs_) from +40 V to −40 V and back to +40 V, at a constant V_ds_ = 2 V drain bias (Figure 2(c)). The results confirm an n-type conduction in the multilayer SnS_2_, consistent with previous findings for flakes of similar thickness.^25^ Notably, complete transistor turn-off is observed only at 80 K, indicating a strong temperature dependence of the threshold voltage. The threshold voltage (V_th_) is obtained from a linear fitting of the transfer characteristic in the on-state.^26^ At 80 K, we estimated V_th_ = (−5.75 ± 0.25) V and the subthreshold swing, $\mathrm{SS}=\frac{{(dV}_{\mathrm{gs}})}{\left(d\log I_{d}\right)}\approx 5.2\frac{V}{\mathrm{decade}}$ . A pronounced hysteresis in the transfer characteristic, with a clockwise direction as indicated by the blue arrows in Figure 2(c), is evident at all measured temperatures. Hysteresis, commonly reported in TMDs-based FETs, arises from a shift in the transfer characteristic during the gate voltage sweep, caused by additional gating from continuous and slow filling and emptying of charged trap states.

**Figure 2:**
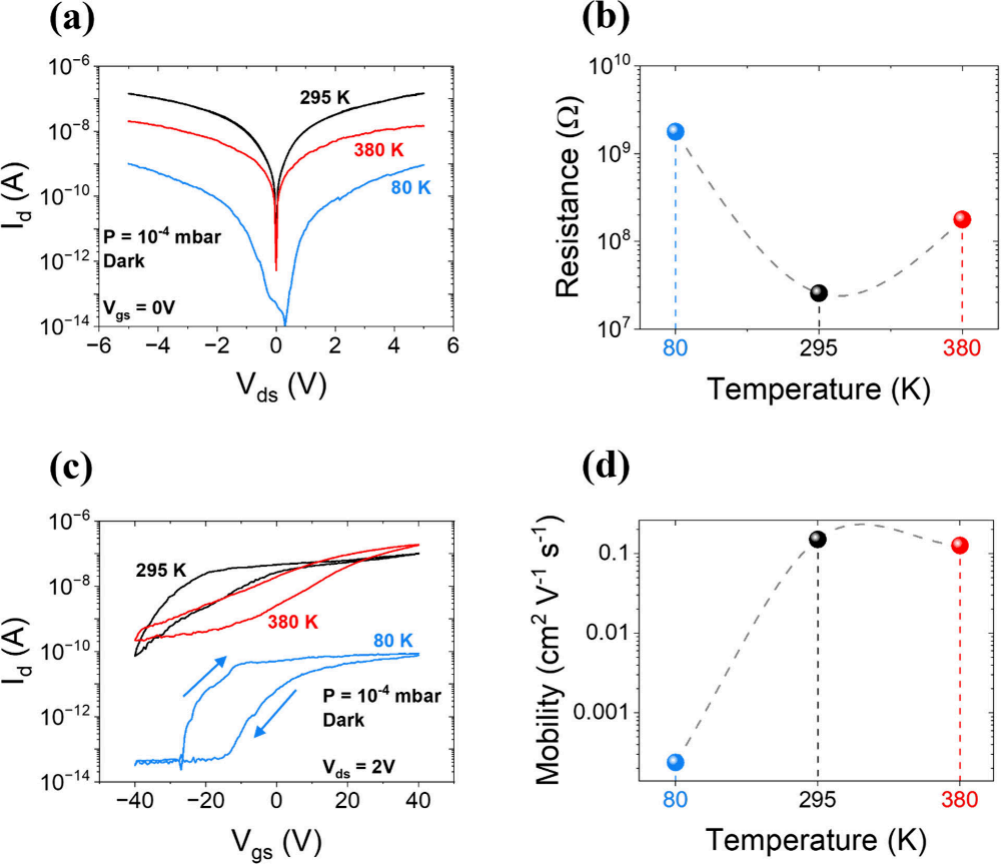
(a) IV curves on semilog scale at *P* = 10^–4^ mbar, with grounded gate and in the dark at *T* = 80 K (blue curve), 295 K (black curve), and 380 K (red curve). (b) Resistance profile extracted from (a) as a function of temperature. (c) Transfer curves in semilog scale, measured at V_ds_ = 2 V, obtained at *T* = 80, 295, and 380 K (same color scheme as in (a)). The blue arrows indicate the sweep direction of the 80 K curve, which is analogous to the other curves. (d) Mobility profile, in semilog scale, extracted from (c) as a function of temperature.

These states are introduced by intrinsic or interfacial defects, such as those originating from process residues, remaining adsorbates, SnO_2_ formation, and interactions with SiO_2_.^12,27^ The clockwise direction of the hysteresis loop suggests the donor-like nature of these traps, which become positively charged when depleted of electrons at negative gate voltages, thus causing a left shift of the transfer curve. This is confirmed by transfer curves shown in Figure S2, acquired at various V_gs_ ranges, showing that the hysteresis width increase is mainly due to a negative shift in the forward branch.^28^ Moreover, the temperature behavior of the channel resistance extracted from the transfer characteristic shows a strong dependence on the applied gate voltage. At V_gs_ = 0 V, the resistance trend is consistent with the profile of Figure 2(b) (R_80 K_ < R_380 K_ < R_295 K_), extracted from the IV characteristics. At V_gs_ = 40 V, an increased density of conduction electrons at elevated temperatures results in higher drain current at 380 K compared to lower temperatures. Likewise, at V_gs_ = −40 V, the increase of SS and decrease of V_th_ with temperature^29^ lead to an increasing I_d_ with the rising temperature. The on/off ratio, defined as I_d_ (V_gs_ = +40 V)/I_d_ (V_gs_ = −40 V), decreases with the increasing temperature, ranging from nearly 2700 at 80 K, to 1400 at 295 K, and reaching approximately 850 at 380 K. Finally, based on the transfer characteristics, the field-effect mobility μ of the majority carriers was estimated through the following expression:^26^

1 $$\mu =\frac{1}{C_{ox}V_{ds}}\frac{L}{W}{\left|\frac{\partial I_{d}}{\partial V_{gs}}\right|}_{Lin.}$$

where C_ox_ = 12.11 nF cm^–2^ represents the SiO_2_ dielectric capacitance per unit area, and ${\left|\frac{\partial I_{d}}{\partial V_{gs}}\right|}_{Lin.}$ denotes the absolute value of transconductance extracted in the transistor linear regime. The resulting mobility exhibits a nonmonotonic temperature dependence, shown in Figure 2(d), with a peak value of 0.15 cm^2^ V^–1^ s^–1^ at T = 295 K and a minimum of ∼ 2 × 10^–4^ cm^2^ V^–1^ s^–1^ at T = 80 K. This behavior, consistent with previous reports on the same material,^12^ suggests the coexistence of two distinct transport-limiting mechanisms. At low temperatures, scattering from ionic impurities is the dominant mechanism, while at higher temperatures electron–phonon interactions dominate, leading to reduced carrier mobility as temperature increases.

### Optoelectronic Characterization

The device photoresponse was then characterized under monochromatic laser illumination. The flake was exposed to light with wavelengths ranging from 420 to 800 nm, in 20 nm steps, and for each wavelength, the drain current was acquired over time. The I_d_ vs time curves (I–t) were first measured in vacuum at T = 295 K with V_ds_ = 2 V and V_gs_ = 0 V, as shown in Figure 3(a). Illumination started at t = 50 s and lasted 100 s, causing an increase in the drain current. We define I_dark,pre_ as average drain current before illumination, I_light_ as average drain current during illumination and I_dark,post_ as the stabilized current after switching the laser off. These quantities are listed in Figure S3 in the Supporting Information. I_light_, shown as colored dots in the I_d_-Wavelength plane of Figure 3(a), reveals a device photoresponse peak at 480 nm. Note that, when the laser is switched on, the drain current reaches an excited steady state rather quickly, with a timing that will be discussed later. To investigate the dominant photogeneration mechanisms in various regions of the spectrum, five wavelengths (480, 520, 560, 660, 740 nm) in the analyzed spectrum were selected. For each, I–t was measured at different levels of the laser power. Results are reported in Figure S3. Photocurrent (I_ph_), defined as I_ph_ = I_light_ – I_dark,pre_, was extracted from each curve, and the I_ph_ vs incident power (P_λ,inc_) data, shown in Figure S4, were fitted with a power law:

2 $$I_{ph}=k{\left(P_{\lambda ,inc}\right)}^{\alpha }+c$$

where P_λ,inc_ denotes the laser power at a given wavelength, incident on the exposed area A of the flake, and k and c are fitting constants. The obtained exponent α as a function of wavelength is reported in Figure 3(b). A decrease of α with increasing wavelength is observed. An exponent α = 1 indicates ideal electron–hole pairs photogeneration, separation and collection.^30,31^ Conversely, an α lower than 1 suggests the involvement of trap-states, which reduce the photocarrier generation rate and lower their collection efficiency by capturing carriers and favoring their recombination.

**Figure 3:**
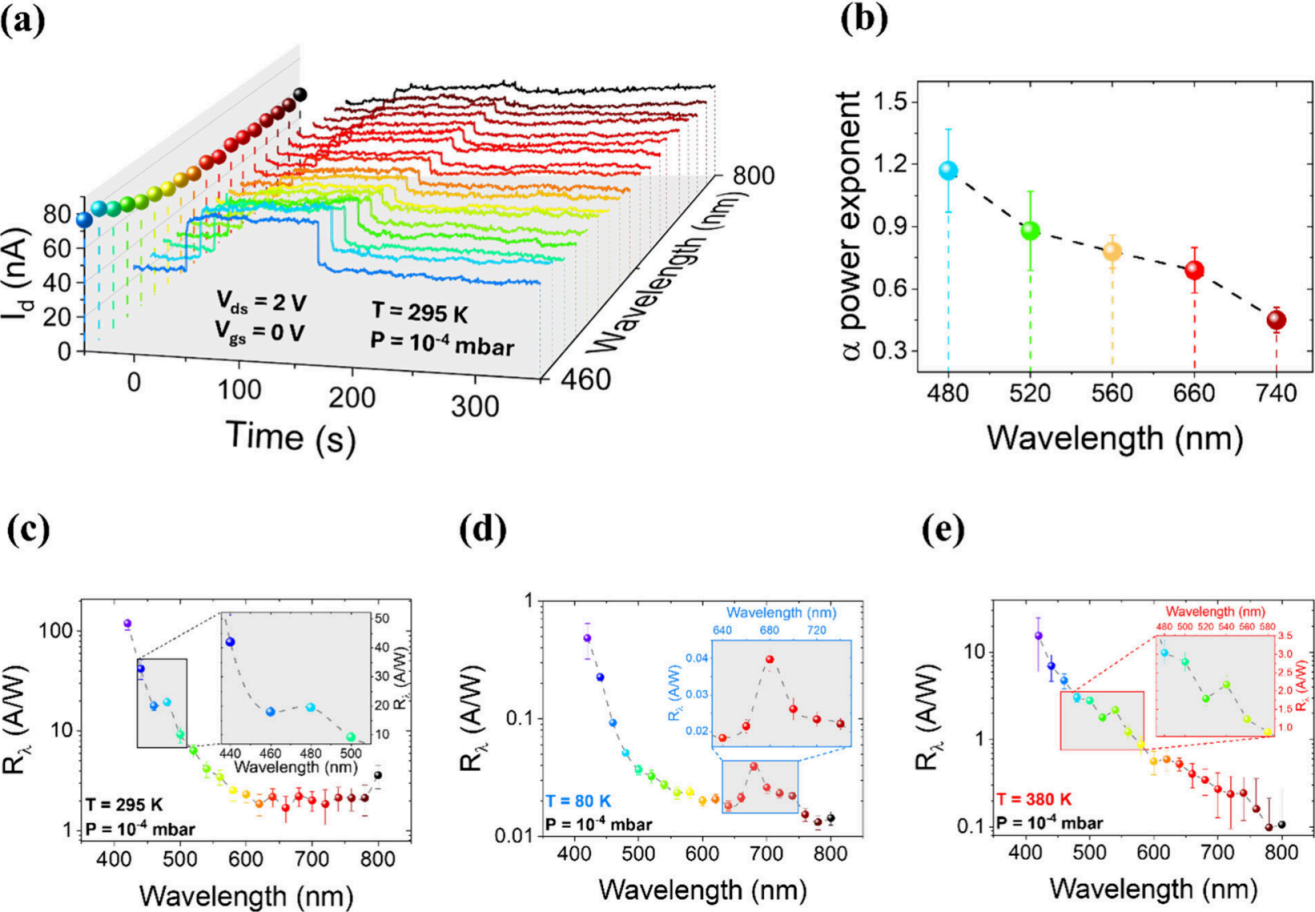
(a) I–t measurements at *T* = 295 K in vacuum under illumination with a monochromatic laser pulse (duration: 100 s), acquired from 460 to 800 nm in 20 nm steps. V_ds_ was set at 2 V and V_gs_ was grounded. The color scale follows the visible electromagnetic spectrum. Colored dots on the I_d_–Wavelength plane indicate average I_d_ values under illumination. (b) Power-law exponent α extracted from photocurrent vs incident power fits, plotted as a function of wavelength. (c–e) Responsivity spectra (semilog scale) as a function of laser wavelength at 295 K (c), 80 K (d), and 380 K (e). Insets: zoomed-in views of spectral peaks.

At 480 and 520 nm, α is consistent with 1, indicating high electron–hole pairs photogeneration and efficient charge collection; conversely, at longer wavelengths, band-to-band transitions are energetically forbidden, so photocurrent could only occur through trap-assisted photoexcitation. The reduction of α from 560 nm onward supports this conclusion, indicating also that there are trap states distributed all across the bandgap.^32^

Afterward, the spectral responsivity spectrum $\left(R_{\lambda }\right)$ , defined as

3 $$R_{\lambda }=\frac{I_{ph}}{P_{\lambda ,inc}}$$

was calculated at T = 295 K. This is shown in Figure 3(c). The maximum responsivity (∼100 A/W) occurs at 420 nm (∼2.95 eV), which lies at a higher energy than the commonly reported direct bandgap of SnS_2_ (∼2.6 eV). While a peak (∼20 A/W) around 480 nm can be associated with the direct L→L transition,^11,33,34^ the enhanced response at shorter wavelengths suggests the involvement of higher-energy optical transitions. Previous studies on multilayer SnS_2_ ^35,36^ have shown that the absorption coefficient increases at photon energies even above the bandgap. In particular, Fan et al.^36^ reported that transitions at the M′ point of the First Brillouin zone, occurring at ∼3.05 eV, may contribute significantly to photocurrent increase at low wavelength. In our case, the responsivity maximum at 420 nm may result from such transitions, possibly assisted by intragap states such as defects or edge-related states that reduce the effective threshold for absorption. The reported responsivities are high and are consistent with, or outperform, those previously reported in the literature for this material.^37,38^

Analogous responsivity spectra were obtained at 80 and 380 K. The resulting spectra are shown in Figure 3(d) and 3(e). At 80 K, a new peak appears at 680 nm, possibly due to high electron density within intragap trap states; indeed, at lower temperatures these traps become more populated. Seeing the wavelength at which this peak occurs, trap states are likely “split interstitial” Sn-related deep donor states at the oxide-semiconductor interface, creating a density of states peak ∼1.7 eV below the conduction band.^39^ However, these are not the only intragap available states, since a nonzero responsivity is also observed for higher wavelengths, ascribable to sulfur vacancies and SnO_2_ formation. Finally, at 380 K the L→L peak shifts to 540 nm (∼2.3 eV); this shift aligns with previous reports of a direct bandgap reduction, of even several hundred meV, in SnS_2_ above 300 K.^40^

### Gate and Temperature Dependent Photoresponse in Vacuum

The next step involved measuring I-t curves at the five previously selected wavelengths and at different V_gs_. This was aimed at probing the role of trap states in conduction since gate voltage modulation allows tuning the occupancy of intragap states, as already observed in discussing transfer characteristics. Figure 4 presents I-t measurements at 295 K in vacuum for gate voltages ranging from −24 V to +24 V, in 8 V steps.

**Figure 4:**
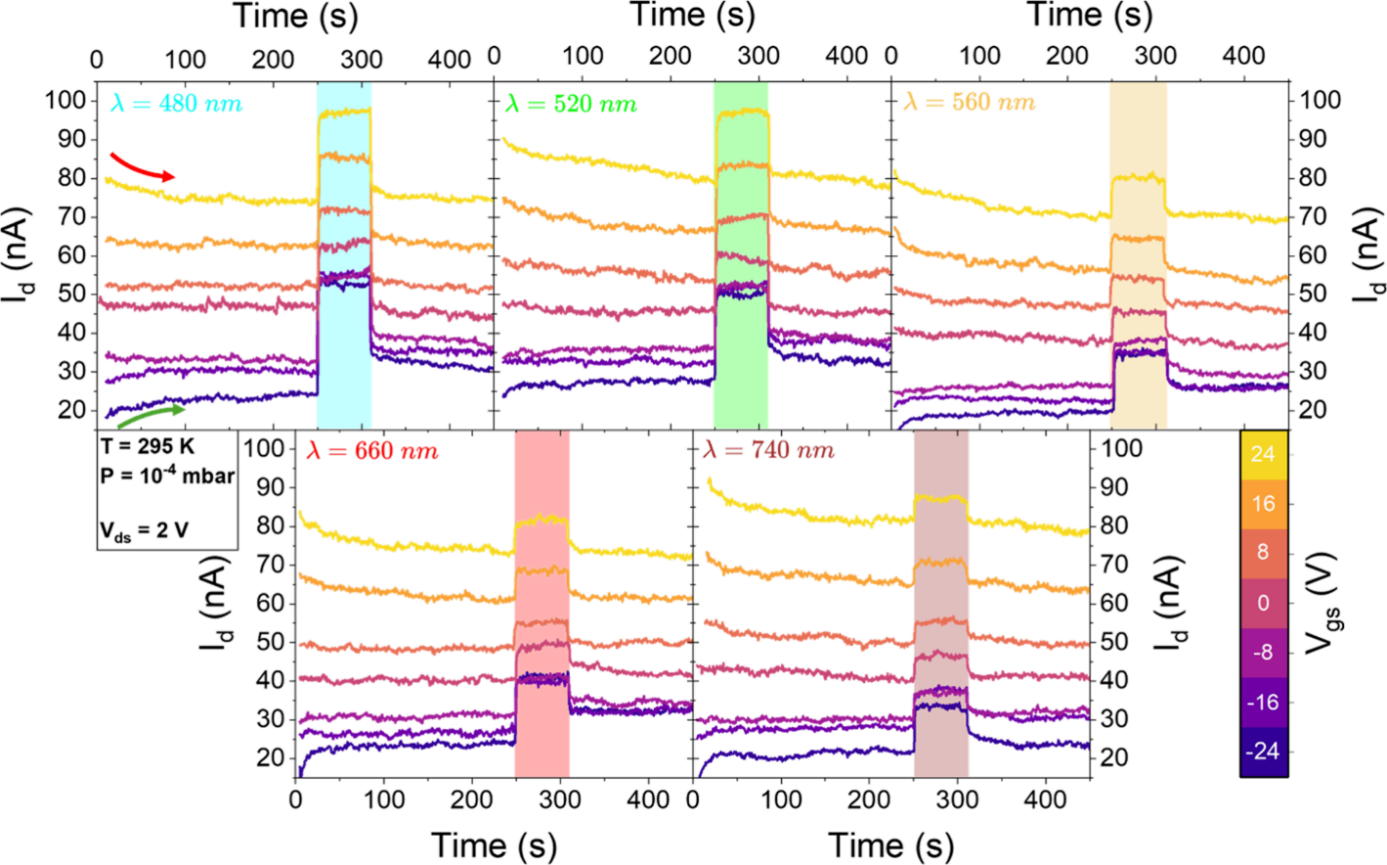
I–t curves acquired at V_gs_ = −24 V (dark blue) to +24 V (yellow), in 8 V steps, for laser wavelengths of 480, 520, 560, 660, and 740 nm. Measurements were performed at 295 K, in vacuum, with V_ds_ = 2 V. The shaded regions in each plot indicate the time intervals during which the laser was on at the specific wavelength.

For each measurement, the gate voltage was applied and held for 250 s before laser activation, allowing the current to stabilize. The laser was then turned on for 60 s, followed by a 150 s postillumination phase. A clear dependence on gate polarity is observed. Negative gate voltages, which deplete donor-like trap states leaving them positively charged, result in initial upward bending (green arrow in top-left panel in Figure 4) in the current profile, while positive gate voltages, which fill traps, lead to downward bending (red arrow in top-left panel in Figure 4).

As previously reported, photogeneration mechanisms in FETs based on 2D materials are various.^32^ To identify those most relevant to the photoresponses observed in Figure 4, both the rising and falling current branches were fitted using a double-exponential function:^41^

$$I_{d}\left(t\right)=I_{0}+A_{1}e^{\left(-(t-t_{0})/\tau_{1}\right)}+A_{2}e^{(-(t-t_{0})/\tau_{2})}$$ 4

where each characteristic time extracted from the fits corresponds to a distinct photogeneration mechanism. Results are reported in Figure 5(a) for the 480 nm case, which is representative of all tested wavelengths. Two characteristic times are consistently observed during both the rise and decay phases: a fast component (τ_2_) on the order of a tenth of a second, and a slower component (τ_1_) on the order of several tens of seconds. These can be attributed to two particular mechanisms in two-dimensional materials. τ_2_ is associated with direct photogeneration, i.e., electron transitions from the valence to conduction, either direct or through intragap states. Although the actual dynamics are expected to occur on submillisecond time scales, the instrumental sampling rate limits time resolution. τ_1_ may be attributed to light-assisted desorption of adsorbates not removed by vacuum,^41^ and mainly to the photogating effect.^42^ The latter involves the excitation of electrons and the depopulation of trap states at the interface with SiO_2_:^41^ a generic trap state of the type described above, denoted as Dn, can be ionized upon illumination through a process of the form Dn → Dn^+^ + *e* ^–^, resulting in the release of a population of electrons that were previously trapped within the channel. The subsequent charging of emptied trap states introduces a local electrostatic potential that modifies the effective gate bias,^43^ In other words, it induces a shift in the threshold voltage ΔV_th_ as a result of charge accumulation Q_trap_ near the oxide-semiconductor interface, which in turn leads to a modification of the flat-band potential. While direct photogeneration is inherently fast, photogating is much slower.^44,45^

**Figure 5:**
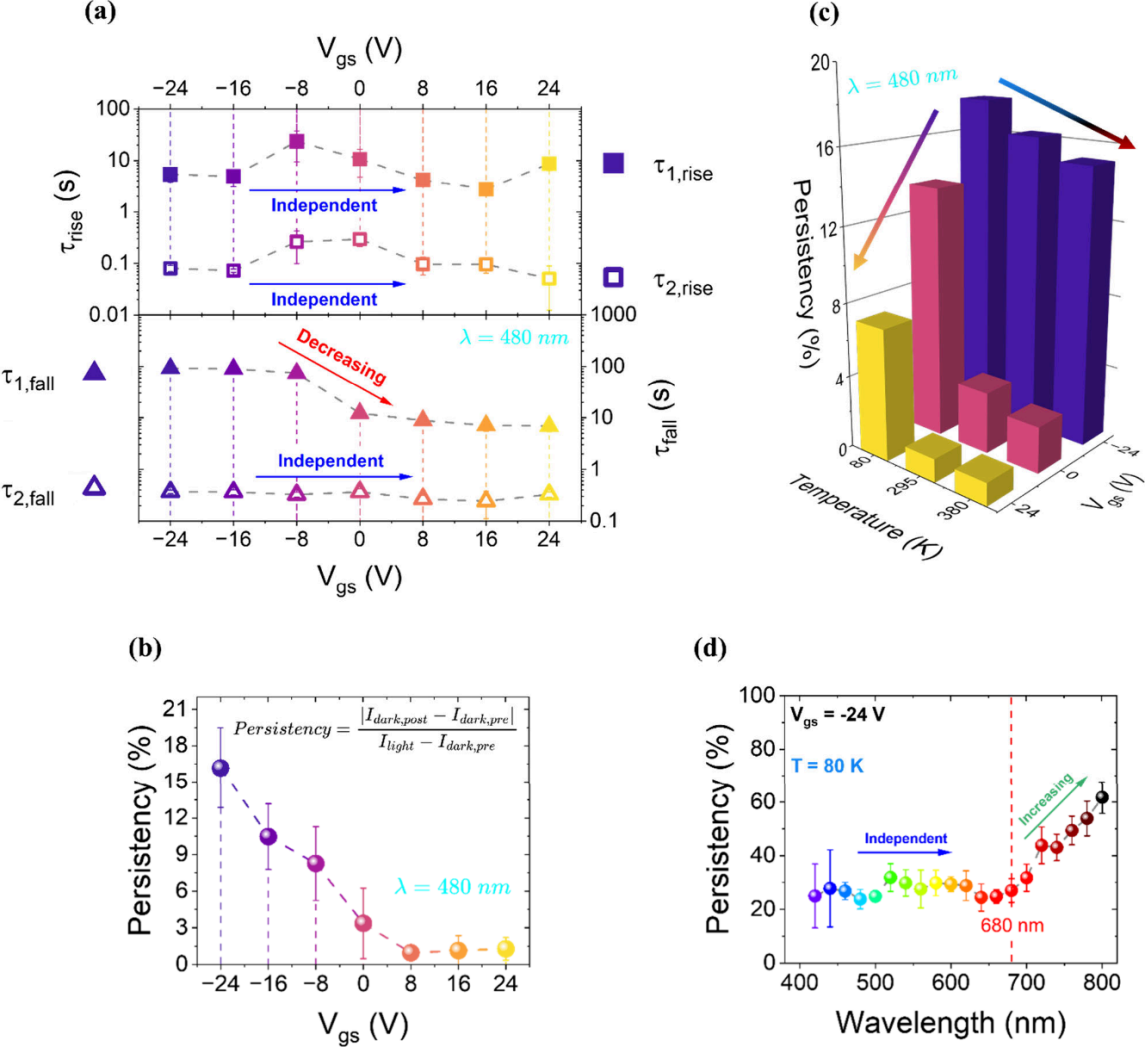
(a) Rise (τ_1,rise_ and τ_2,rise_) and falling (τ_1,fall_ and τ_2,fall_) characteristic times as a function of V_gs_, extracted from I–t measurements at 480 nm (semilog scales). Blue arrows indicate gate-independent behavior; the red arrow highlights a decreasing trend with increasing gate voltage. (b) Persistency (%) as a function of V_gs_ for 480 nm illumination. Inset: definition formula of the Persistency parameter. (c) Histogram of Persistency vs temperature and V_gs_ at 480 nm. Colored arrows represent increasing V_gs_ (from dark blue to yellow) and increasing temperature (from blue to red). (d) Persistency calculated at 80 K and V_gs_ = – 24 V as a function of incident wavelength. The blue arrow indicates wavelength-independent behavior; the green arrow marks an increasing trend of Persistency with longer wavelengths.

The identification of the slower relaxation process as photogating, and the central role of donor-like trap states in this mechanism, is supported by Figure 4. I_light_ shows a clear asymmetry with respect to gate bias. From −24 V to −8 V, at fixed wavelength, the I_light_ value remains relatively constant. In contrast, from 0 V to +24 V, I_light_ increases with gate voltage. This behavior is consistent with the transfer curves under illumination shown in Figure S5­(a). For V_gs_ > V_th_ the device operates in the on state and light-induced carriers enhance conduction, so that the transfer curve has a similar shape as the dark one, only slightly raised. For V_gs_ < V_th_, the device would be in the off state, but light-induced photogating, resulting in trapped positive charges, governs conduction. A kink appears in the curve under illumination, marked by a colored arrow in Figure S6­(a), indicating the transition from carrier photogeneration in the on state to a photogating regime in the off state. As a consequence, below V_th_, the applied V_gs_ has no longer control over the modulation of the channel. Different wavelengths can interact with distinct trap states within the bandgap, leading to wavelength-dependent photogating effects. This is reflected in the variation of I_d_ levels at negative V_gs_ across the light-induced transfer curves shown in Figure S6­(a). The lack of hysteresis further supports this interpretation: once the trap states are emptied by illumination and the slow trapping/detrapping mechanism ceases, the system exhibits no hysteresis.

Another observed phenomenon is that, for every wavelength, the drain current, after light is turned off, releases to a level which can be different from the starting one and dependent on the V_gs_. As the gate becomes more negative, I_dark,post_ deviates increasingly from the initial dark current I_dark,pre_, indicating the presence of persistent photoconductivity, an effect previously reported in other two-dimensional materials.^46,47^ Persistent photoconductivity arises from the slow reoccupation of trap states by carriers once the illumination is turned off and the system begins to relax toward equilibrium. As reported in the literature, this slow recovery is attributed to a potential energy barrier associated with the trap states, which originates from the outward structural relaxation of the Dn sites surrounding bonds upon ionization.^48^ This barrier hampers the reneutralization of the trap states, thereby prolonging the photoconductive response. This is highlighted in Figure S6­(b). Notably, our results suggest that this persistence can be modulated via gate voltage, enabling potential applications, as discussed later. In Figure 5(a), this current persistency, associated with the photogating effect, manifests as a larger τ__1_,fall_, which becomes gate-dependent. Trap states are obviously involved in this phenomenon through delayed or suppressed reoccupancy after exposure to light.^49^ In contrast, the τ__1_,rise_ time, related to trap emptying under illumination, appears independent of gate voltage.

To quantify the photocurrent persistence, we introduce the “Persistency” parameter, defined as

$$Persistency\;\left(\%\right)=\frac{\left|I_{dark,post}-I_{dark,pre}\right|}{I_{light}-I_{dark,pre}}(\%)$$ 5

which ranges from 0% to 100%: 0% is for no persistence (I_dark,post_ = I_dark,pre_), and 100% for full persistence (I_dark,post_ = I_light_). As shown in Figure 5(b), the Persistency strongly depends on gate voltage. It reaches a maximum of approximately 15% at V_gs_ = −24 V and decreases with increasing V_gs_, becoming negligible (near 0%) from V_gs_ = 0 V onward.

To confirm that this effect arises from the slow electron retrapping, additional I-t measurements were carried out at 80 and 380 K for varying V_gs_. Temperature plays a critical role in this process: at high temperatures, the carrier density in the channel is higher, which accelerates the retrapping process after the light is switched off; conversely, lower temperatures suppress trap state refilling. Figure 5(c) presents a histogram of Persistency values for V_gs_ = −24 V, 0 V, and +24 V at 80, 295, and 380 K under 480 nm illumination. At all temperatures, Persistency increases as gate voltage becomes more negative, as already observed. More importantly, at fixed gate voltage, Persistency decreases with increasing temperature. This is because at elevated temperatures a higher density of electrons in the channel facilitates electron trapping which results in decreased persistence in the current. Temperature thus emerges as a key parameter, alongside gate voltage, for tuning the duration of postillumination conduction.

The role of wavelength was also examined. Figure S7 displays transfer characteristics in the dark and under illumination at five wavelengths, measured at 80 and 380 K, respectively. At 80 K, a significantly stronger photogating effect is observed. At this temperature, most trap states are filled. Excitation of trapped electrons under light causes the formation of localized positive charge, leading to a substantial gating effect that keeps the transistor in the on state. Figure 5(d) shows the full wavelength-dependent Persistency spectrum at 80 K. Persistency remains approximately constant (∼30%) up to 680 nm, the wavelength corresponding to the previously identified trap state peak. Beyond this point, Persistency gradually increases. This behavior suggests that up to 680 nm, a high density of trap states is efficiently excited. At longer wavelengths, fewer traps are activated and fewer electrons are released, slowing the retrapping dynamics and enhancing persistence.^41^

The gate dependence of the retrapping mechanism under vacuum conditions, when the trap states at the oxide-semiconductor interface are the dominant ones, could be qualitatively explained as follows. When a negative gate voltage is applied (Figure 6(a)), the Fermi level of the gate is raised by an energy eV_gs_ relative to equilibrium. This creates a potential drop across the gate oxide and induces band bending in both the oxide and the SnS_2_ layer near the interface. As a result, an electric field is established, pointing from the SnS_2_ toward the oxide. Upon laser illumination, trap states are emptied, and electrons are excited into the conduction band. However, the local electric field at the interface causes these electrons to drift away from the oxide interface (Figure 6(b)), effectively reducing the local electron density in that region.^29^

**Figure 6:**
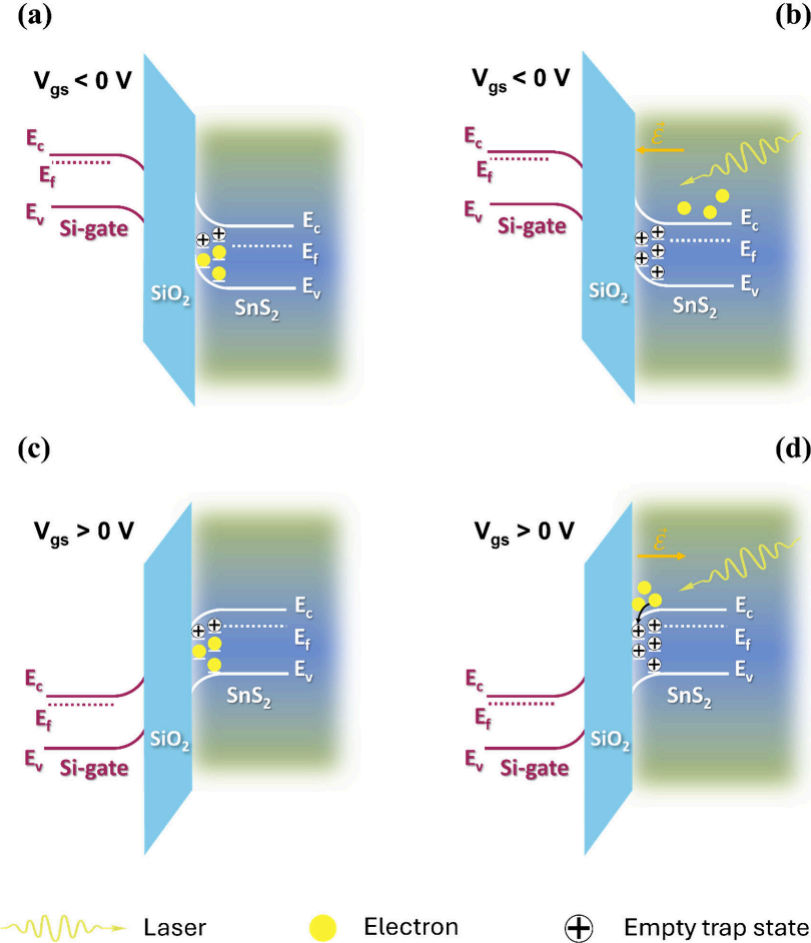
Band diagrams illustrating the mechanism of current persistence in I–t measurements at V_gs_ = −24 V with the laser off (a) and on (b), and at V_gs_ = +24 V with the laser off (c) and on (d). *ε⃗* is the electric field near the interface.

This spatial depletion does inhibit the neutralization of ionized donor-like trap states Dn^+^+ e^–^→ Dn, so that *Q*_*trap*_ remains high, as does the ΔV_th_. In other words, the retrapping dynamics is, once the laser is switched off, much slower, resulting in longer relaxation times and persistent photoconductivity. Conversely, under a positive gate voltage (Figure 6(c)), the gate Fermi level is lowered, reversing the band bending. The electric field now points from the oxide toward the SnS_2_. In this configuration, photogenerated electrons are attracted toward the interface (Figure 6(d)), increasing their local concentration near the trap states. Moreover, the local electric field also affects the energy barrier that inhibits the neutralization of donor states, effectively modulating its shape. By bending the barrier profile, the field facilitates the reoccupation of these states once the illumination is turned off, leading to a rapid decay of the photocurrent and suppression of the persistent response.

In summary, both temperature and V_gs_ are key parameters for tuning current persistence, as both affect the density of electrons in the channel near the SnS_2_/SiO_2_ interface, where most of the trap states are located and thus the retrapping dynamics after the light is turned off.

### Gate and Temperature Dependent Photoresponse in Air

The final step involved electrical characterization in ambient conditions. It is well established that atmospheric species, such as molecular oxygen and water, influence the photoresponse of two-dimensional materials due to their high surface-to-volume ratio.^50^ SnS_2_ is not an exception and the presence of SnO_2_, that is a polar material, on its surface enhances the interaction with polar molecules like H_2_O, and even with relatively nonpolar O_2_ via weak dipole-induced interactions.^51^ Figure 7(a) shows the dark IV characteristics at 295 K, at ambient pressure and at different wavelengths. Compared to the vacuum measurement in Figure 2(a), the curve displays a pronounced asymmetry. This can be attributed to the formation of nonzero and asymmetric Schottky barriers at the Cr/SnS_2_ interfaces, which were negligible under vacuum. Similar effects have been observed in other 2D materials, such as WS_2_.^52^ Adsorption of atmospheric species onto the material surface, and especially at sulfur vacancies, induces local charge accumulation. This leads to Fermi level pinning and nonzero Schottky barriers, which can result in different barrier heights at the two electrodes and asymmetric IV characteristics.^53^

**Figure 7:**
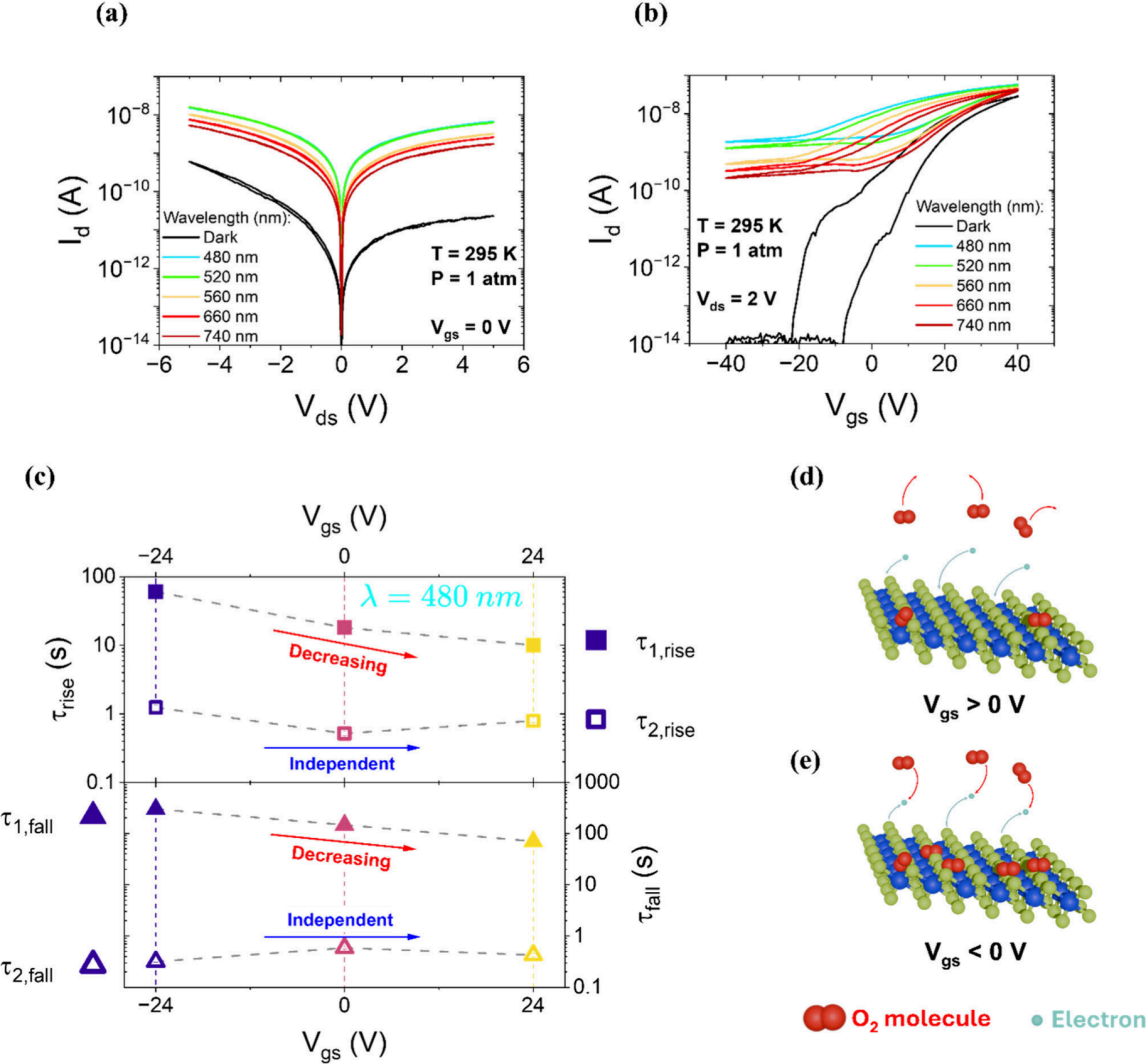
(a) IV characteristics on semilog scale at *T* = 295 K and ambient pressure with grounded gate, measured both in dark (black curve) and under illumination (colored curves). (b) Transfer curves at *T* = 295 K, ambient pressure, and V_ds_ = 2 V, acquired in vacuum (black curve) and under illumination (colored curves). (c) Rise (τ_1,rise_, τ_2,rise_) and falling (τ_1,fall_, τ_2,fall_) relaxation times as a function of V_gs_, extracted from I–t measurements at 480 nm (semilog scales). (d–e) Schematic illustration of the light-induced desorption mechanism under positive (d) and negative (e) gate voltages.

Adsorption of oxygen in particular, due to its high electronegativity, means the capture of a conduction electron from the channel by the mechanism O_2_ + *e* ^–^ → O_2_^–^. From an energy standpoint, given the reported adsorption energy *E*_ads_ of oxygen on SnS_2_ (ranging from −0.4 to −1.9 eV depending on sulfur vacancy concentration),^54^ oxygen can form states in the bandgap, specifically at energy *E*_ads_ below the conduction band. These states, filled by conduction electrons, become negatively charged. Upon illumination, these trap states are emptied, molecular oxygen goes back to zero charge according to O_2_^–^ → O_2_ + *e* ^–^, and desorbs, restoring contact symmetry. This is confirmed by the symmetric IV curves under illumination across all wavelengths, as shown in Figure 7(a) (and in Figure S8 on linear scale in Supporting Information). Moreover, the oxygen adsorption process introduces p-type doping, reducing the overall conduction electron concentration compared to vacuum conditions.

Supporting evidence is provided by the transfer curve in the dark (Figure 7(b)). The oxygen-induced p-type doping allows the device to be switched off, with an on/off ratio nearly 7 orders of magnitude and a V_th_ = (25.75 ± 0.25) V, significantly higher than under vacuum and comparable to the best results in literature.^55^ The field-effect mobility is reduced to 0.05 cm^2^ V^–1^ s^–1^, likely due to enhanced carrier scattering from adsorbed species. A pronounced photogating effect is still observed, as evidenced by the flattening of transfer curves under illumination. As in vacuum, I-t measurements were conducted by illuminating the sample with monochromatic laser light from 420 to 800 nm in 20 nm steps. The resulting responsivity spectrum, shown in Figure S9, reveals a peak at 480 nm, consistent with electron–hole pair photogeneration previously identified under vacuum.

Subsequently, I–t measurements were performed at 480 nm under varying gate voltages (V_gs_ = −24, 0, and +24 V). The experimental results, acquired with the same timing protocol used in vacuum, are shown in Figure S10. From these measurements, the relaxation times reported in Figure 7(c) were extracted. As in vacuum, two distinct characteristic times are observed in both the rising and falling edges of the signal: a fast component τ_2_, attributed to photogeneration, and a slower component τ_1_.

However, a key difference emerges under ambient conditions: both τ_1,rise_ and τ_1,fall_ exhibit strong gate dependence and reach significantly higher values than those measured in vacuum, often on the order of hundreds of seconds. This increase is attributed to the coexistence of two simultaneous light-related mechanisms: one intrinsic, due to interface trap states (already active in vacuum), and a second, extrinsic, arising from adsorbed atmospheric species, primarily oxygen molecules. These adsorbates capture conduction electrons from the material and become negatively charged, acting as a p-type dopant. Upon illumination, they undergo photoinduced desorption, releasing the captured charge and contributing to the photocurrent. The gate dependence of the rise time in air can be understood by examining how the gate-induced electric field modulates the electron density near the surface, which in turn controls the effectiveness of oxygen adsorption and desorption. Oxygen adsorption is most efficient when conduction electrons are available at the surface, as the molecule needs to capture an electron to become stabilized. When a positive gate voltage is applied (Figure 7(d)), the electric field draws electrons toward the oxide interface, depleting the surface of available carriers. In this condition, adsorbed oxygen molecules are more easily desorbed upon illumination due to the lack of surface-bound electrons that could stabilize them. This facilitates a faster rise in photocurrent. Conversely, when a negative gate voltage is applied (Figure 7(e)), the electric field pushes electrons toward the top SnS_2_ surface, increasing the density of conduction electrons available for oxygen adsorption. In this case, adsorbed oxygen molecules are more stable and desorption under illumination is less efficient, resulting in a slower photocurrent rise.

Once the light is turned off, oxygen molecules slowly readsorb onto the surface, gradually capturing electrons again and restoring equilibrium. Similarly to the case of donor-like trap states, the readsorption of oxygen molecules after the illumination is switched off does not occur instantaneously. This process is also hindered by an energy barrier that must be overcome for an oxygen molecule to capture an electron and become chemisorbed onto the material’s surface, a mechanism that contributes to persistent photoconductivity. As a result, in ambient conditions, persistent photoconductivity arises from the combined effects of both interfacial trap states and oxygen readsorption. Like in vacuum, this dual mechanism is expected to show gate dependence, which is indeed confirmed by our measurements. However, due to differences in the energetic landscape of the two processes, the adsorbate-mediated mechanism is inherently slower than trap-state recombination and therefore tends to dominate the long-term relaxation dynamics under specific gate bias conditions.^56^ The previous model proposed in Figure 6 can now be expanded by also taking into account the adsorbates: during the decay phase, the two relaxation pathways, i.e., trap-state recombination and oxygen readsorption, proceed simultaneously. Depending on the gate polarity, either mechanism can dominate the decay dynamics: under negative gate bias, when electrons are pushed toward the surface, the interface-related trap dynamics is hindered and the readsorption of oxygen molecules is facilitated. This means that the slower trap-related process becomes the rate-limiting step. This explains the minor change that τ_1,fall_ has at −24 V, going from ∼100 s in vacuum to ∼ 200 s in air. Conversely, under positive gate voltage, electrons are drawn toward the oxide interface and depleted from the surface. In this case, the trap-state recombination is relatively efficient, but the oxygen readsorption becomes extremely slow due to the lack of available electrons at the surface. As a result, the adsorbate-mediated mechanism becomes the dominant relaxation channel, causing a much longer decay time in air compared to vacuum. For this reason, τ_1,fall_ increases from ∼7 s in vacuum to ∼80 s in air at +24 V, showing that oxygen readsorption now dictates the time scale.

### Field-Effect Transistor as a Synapse

The gate-tunable photoresponse of SnS_2_ suggests potential for neuromorphic applications. To understand this connection, a basic overview of neuronal signaling is provided. Neurons, shown in Figure 8(a), are excitable cells that communicate through electrical impulses called action potentials.^57^ At rest, a neuron maintains a potential difference with respect to extra-cell environment of approximately −70 mV due to the selective permeability of its phospholipid membrane and the activity of ion pumps. These regulate the intracellular and extracellular concentrations of ions like Na^+^, K^+^, and Ca^2+^. External stimuli, such as light at the retinal level, are detected by sensory receptors, which trigger the release of neurotransmitters. These chemical messengers bind to receptors on adjacent neurons, modulating the opening or closing of ion channels. If the resulting ion flux to the neuron depolarizes the membrane beyond a threshold voltage, an action potential is initiated. This spike (Figure 8(a)) involves a rapid rise in membrane potential (to about +40 mV), followed by repolarization and a brief hyperpolarization before returning to baseline. This is action potential. Signal propagation between two neurons occurs at synapses, i.e., the junctions between the axon (the end piece of a neuron) of a presynaptic neuron and the dendrites (the initial part of a neuron) of a postsynaptic neuron. When an action potential reaches the axon terminal, it induces the release of neurotransmitter-containing vesicles. These neurotransmitters bind to ligand-gated ion channels on the postsynaptic membrane, modulating its potential. If depolarization is sufficient, a new action potential is triggered in the postsynaptic neuron. This represents the basic mechanism of excitatory synapses, which increase the likelihood of spike generation in the postsynaptic neuron. In contrast, inhibitory synapses reduce this likelihood by hyperpolarizing the membrane, making action potential initiation less probable. Synapses not only transmit signals but also modulate them. Their strength, or so-called synaptic weight,^58^ refers to the amplitude of the connection between two neurons, which is the capacity to generate a stronger or weaker postsynaptic spike or to retain it. This can vary over time in response to activity and other conditions. This phenomenon is known as synaptic plasticity. This includes both potentiation and depression of the postsynaptic response. Synaptic plasticity is the biological basis of learning and memory. Neuromorphic systems aim to replicate these mechanisms in hardware.

**Figure 8:**
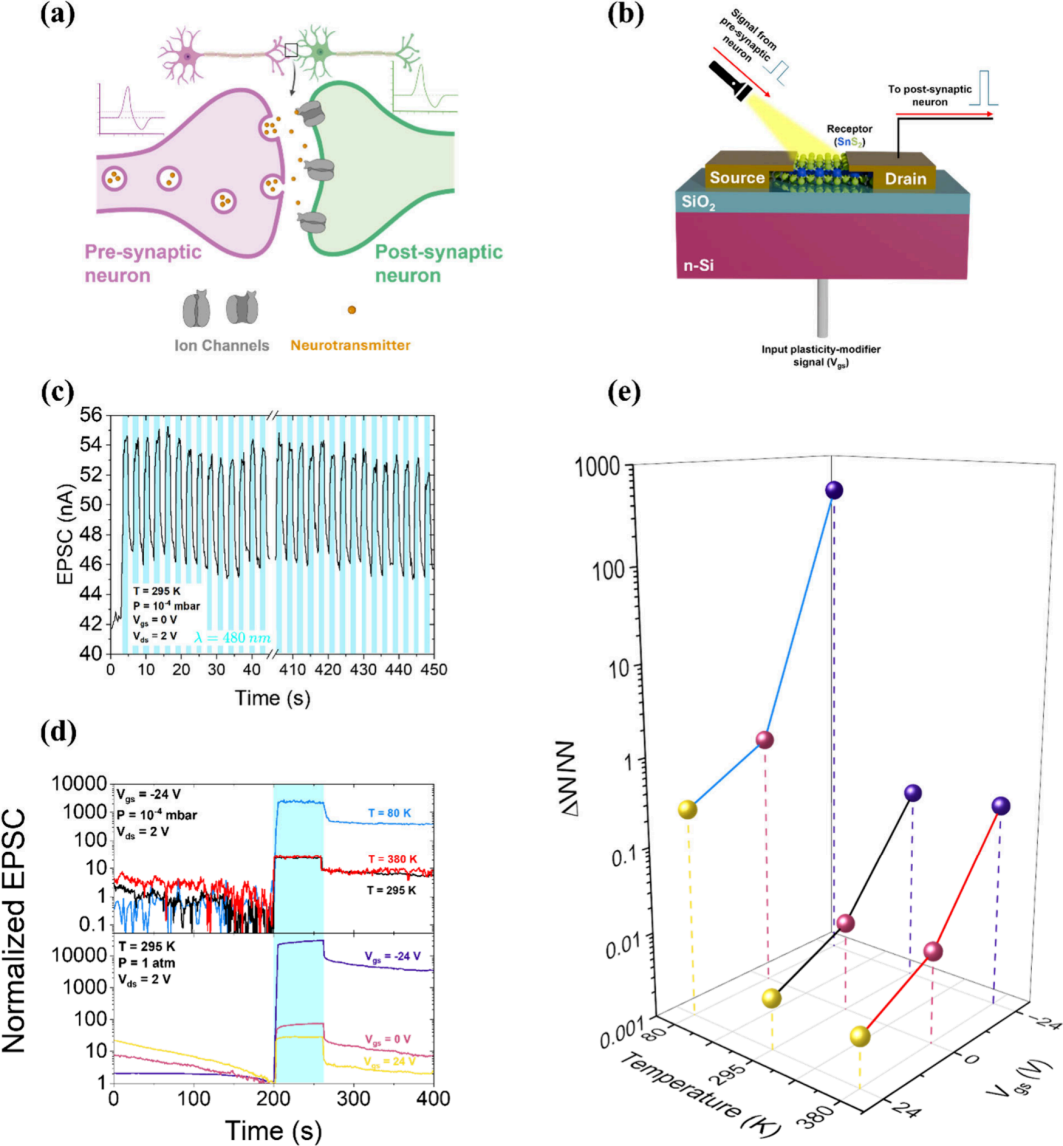
(a) Schematic of a biological synapse between pre- and postsynaptic neurons, illustrating ion channels and neurotransmitter release. A typical action potential profile is also shown. (b) Conceptual representation of the device functioning as an artificial synapse. (c) EPSC response under repeated presynaptic stimuli (150 pulses, 480 nm pulses, 295 K, vacuum). (d) EPSC profiles in vacuum at various temperatures (top) and in air at various V_gs_ (bottom), normalized to the respective prestimulus current. (e) Synaptic weight changes as a function of V_gs_ and temperature, measured in vacuum.

Synaptic plasticity is inherently complex to replicate in hardware, yet the observed behavior of chalcogenide materials offers a promising approach to emulating it. To model neural processes, we map the elements of the SnS_2_ FET as illustrated in Figure 8(b). The laser pulse acts as the presynaptic stimulus; the device itself represents the synapse. The 2D material mimics the axon terminal receptor, with conduction electrons serving as neurotransmitters, and the drain current spike mimics the postsynaptic signal. In biological systems, neurotransmitter-induced activation of ion pumps alters the membrane potential, triggering a voltage spike in the postsynaptic neuron. Analogously, in our device, the optical excitation of electrons induces a photoconductive response, generating a spike in the drain current.

Current spike is therefore functionally equivalent to an Excitatory Post-Synaptic Current (EPSC). The EPSC propagates to the drain electrode, representing signal transmission to a postsynaptic neuron. Importantly, after the light is switched off, the drain current does not immediately return to baseline but remains elevated due to persistent photoconductivity. This retention of current mimics postsynaptic excitatory memory of the presynaptic input, i.e., plasticity. Additionally, the gate voltage acts as a tuning parameter, modulating the density of photogenerated electrons (neurotransmitters) and thus controlling the amplitude of the EPSC after presynaptic spike. This enables modulation of the synaptic weight, i.e., the strength of the postsynaptic response.

To merge and formalize the whole, synaptic weight can be quantitatively defined as the transistor conductance:^59,60^ specifically, W_0_ = I_dark,pre_/V_ds_ denotes the synaptic weight (i.e., the conductance of the transistor) prior to the application of the presynaptic pulse, while W_1_ = I_dark,post_/V_ds_ represents the synaptic weight after the pulse has been applied. In general, W_i_ indicates the synaptic weight after the i-th presynaptic impulse. Moreover, it is interesting to evaluate how synaptic weight changes after one presynaptic pulse, which is necessary to estimate synaptic plasticity: synaptic weight change ΔW/W is defined as the relative change of synaptic weight induced by a presynaptic pulse (laser excitation), following the method outlined in^61^

6 $$\frac{\Delta W}{W}\equiv \frac{W_{1}-W_{0}}{W_{0}}=\frac{I_{dark,post}-I_{dark,pre}}{I_{dark,pre}}$$

When this is zero, the postsynaptic signal mirrors the presynaptic input precisely, ceasing as soon as the stimulus ends. Small values mean short-term plasticity, whereas high values mean long-term plasticity. As it increases, it indicates memory retention, with the postsynaptic current sustained beyond the stimulus duration.

The change in synaptic weights under the application of multiple consecutive presynaptic pulses was monitored: Figure 8(c) shows EPSC versus time measurements under repeated 480 nm light pulses (1.5 s on, 1.5 s off). Unlike other materials reported in literature,^61^ SnS_2_ device seems to exhibit no cumulative EPSC enhancement under repeated stimulation, indicating the absence of activity-dependent plasticity. This is further confirmed by Figure S11 in the Supporting Information, which shows both the evolution of synaptic weights and changes in synaptic weights at successive presynaptic impulses. This behavior allows precise control of synaptic output via a single parameter without unintended memory accumulation after more than one presynaptic input. However, we do not exclude the possibility that a cumulative EPSC effect could emerge if the interval between successive light pulses were further reduced. We note, however, that standard benchmarks such as spike-timing-dependent plasticity (STDP) and paired-pulse facilitation (PPF) were not assessed in this work due to limitations in our current instrumentation. These features typically require submillisecond time resolution that exceeds the capabilities of our present setup. Nonetheless, the observed gate- and temperature-tunable EPSC behavior, combined with stable responses under repeated stimulation shown in Figure 8(c), confirms the suitability of SnS_2_ FETs as candidate artificial synapses.

On the other hand, to emphasize the tunability of the EPSC, i.e., of the synaptic strength, via V_gs_ and temperature, Figure 8(d) shows time-resolved EPSC profiles normalized to the prestimulus dark current, i.e., (I_d_ – I_dark,pre_)/I_dark,pre_. From a temperature perspective (Figure 8(d), top), at a fixed gate voltage of −24 V, the postsynaptic current exhibits increased persistence as temperature decreases. Specifically, the EPSC remains approximately 400 times higher than the prestimulus baseline at 80 K, compared to only about 10 times higher at 295 and 380 K, which corresponds to longer-term plasticity as temperature decreases. Conversely, at a fixed temperature (Figure 8(d), bottom), increasing the magnitude of the negative gate voltage enhances EPSC retention following the presynaptic pulse. At −24 V, the postsynaptic current reaches values nearly 3000 times greater than the prepulse level, while dropping close to unity for 24 V.

From the persistence data in Figure 5(c), the synaptic weight changes ΔW/W can be extracted as a function of gate voltage and temperature, as shown in Figure 8(e). Gate voltage, in particular, shows strong modulation capability, enabling continuous tuning of synaptic strength, and from short to long-term plasticity, a key for controlling memory-related behavior. A maximum ΔW/W of ∼500 is recorded at 80 K and V_gs_ = −24 V in vacuum, indicating that the postsynaptic current stabilizes at a value approximately 500 times higher than its prestimulus (dark) level, persisting over time, indicative of long-term plasticity. These results appear comparable and even better than similar devices in the literature also based on other materials.^61–63^

Under ambient pressure and temperature conditions, the device exhibits even better performance. As shown in Figure 8(d), it is possible to estimate a value of ΔW/W of approximately 2 at V_gs_ = 24 V, around 8 at 0 V, and exceeding 3000 at −24 V. These results highlight not only the potential for more effective long-term plasticity compared to vacuum conditions, but also a high degree of tunability. Moreover, it is important to emphasize that cryogenic vacuum measurements were performed solely to isolate the intrinsic dynamics of the SnS_2_ trap state, while all key neuromorphic metrics, including gate-tunable EPSC retention, synaptic weight variations spanning 3 orders of magnitude, and cycle endurance, were demonstrated at room temperature in ambient air, confirming the practical applicability of the device under realistic conditions.

Finally, the relevance of our results is underscored by benchmarking them against state-of-the-art neuromorphic devices based on two-dimensional materials reported in the literature; the most pertinent examples are summarized in Table 1.

**Table 1:** Benchmarking Synaptic Weight Change of Our Two-Dimensional Neuromorphic Device against Representative State-of-the-Art Counterparts Reported in the Literature

2D Material/Heterostructure	Device Type	Measurement Conditions (environment, light stimulus)	ΔW/W	Reference
MoS_2_ monolayer/SiO_2_	Optoelectronic FET	450 nm light, gate –2 V, room temp	≈9	^64^
MoS_2_/ZnO	Synaptic transistor	Visible light, room temperature, strong PPC	>1	^65^
Graphene/MoS_2_	Mechano-opto-synaptic transistor	Green light (525 nm), triboelectric trigger (TENG), ambient	≫1	^66^
Black Phosphorus (BP)	Optoelectronic synapse	UV/visible light, buck oxide cap, few-layer BP, room temp	<10^3^	^67^
WS_2_/PZT	Optoelectronic memristive transistor	Visible light, multilevel conductance states, ferroelectric PZT	<3000	^68^
MoS_2_/Graphene FG	Floating-gate neuromorphic transistor	Visible light + electric pulses, ambient	<1000	^69^
α-In_2_Se_3_ (ferroelectric)	Bidirectional phototransistor	Visible light, room temp, dynamic optical spikes	<3000	^70^
**SnS**_**2**_	**Optoelectronic synaptic FET**	**Visible light** **(420–800 nm)**, **gate sweep** (−24 V to +24 V), 80–380 K, vacuum and ambient air	**>3000**	**This work**

## Conclusions

In conclusion, in this work, we have provided the first comprehensive, multiparameter investigation of SnS_2_ back-gate field-effect transistors, revealing how the same device can be continuously transformed from a high-gain photodetector into a gate-programmable artificial synapse. By exploring a wide experimental space (temperatures from 80 to 380 K, pressures from high vacuum to ambient air, and illumination from 420 to 800 nm) we have clarified the individual roles of interface trap states and adsorbed oxygen molecules in determining the persistent photoconductivity that underlies synaptic behavior. At room temperature under vacuum, the transistor offers a record sensitivity of approximately 100 A/W at 420 nm and a synaptic weight variation ΔW/W approaching 500, values that establish the intrinsic upper limit of the material. At 295 K in air, where practical neuromorphic hardware must ultimately operate, the same structure maintains broadband photoresponsivity, exhibits fully reversible EPSC peaks over hundreds of light pulse cycles, and, thanks to the interaction between interface traps and oxygen-mediated photogating, retains the gate-voltage-induced modulation in the material’s photoresponse. Crucially, commonly undesirable trap states within the bandgap are shown to enable synaptic plasticity, with adjustable synaptic weights changing from 0.001 to near 3000 and controllable transition between short- and long-term behavior.

These findings make SnS_2_ even more attractive and establish a foundation for its use in future neuromorphic architectures that aim to replicate the adaptive properties of biological neural networks.

## Experimental Section

Single crystals of SnS_2_ were synthesized via the chemical vapor transport method. High-purity (better than 99.9%) ingots of Sn and S with a molar ratio of 1:2.02 were mixed together and pressed into a pellet. The pellet, along with a small amount of Iodine (2 mg/cm3), was sealed in an evacuated quartz tube and placed into a two-zone tube furnace. The vapor transport reaction was carried out in a two-zone furnace between 873 K (source) and 803 K (sink) for 10 days. Finally, thin hexagonal-shaped single crystals with shiny surfaces were obtained. Crushed crystals of SnS_2_ were characterized by X-ray powder diffraction with Cu–Kα radiation (Bruker D2 Phaser diffractometer) at room temperature. The single crystal quality and crystallization directions were identified by Laue diffraction (Photonic Science).

A 200 kV JEOL 2200FS TEM in Lund was used to examine the SnS_2_ material. Flakes of the material were stored in isopropanol. A few microlitres were dropped onto a TEM grid; blotting paper under the grid removed the liquid, allowing flakes to adhere to the carbon film on the grid. (The carbon film appears in Figure 1(f) as thin strands supporting the flake). Energy-dispersive X-ray spectroscopy (EDS) was performed on a 300 kV Hitachi H3300 TEM.

Electrical measurements were performed using a Lake Shore probe station, equipped with 25 μm-radius gold-coated tungsten probes, with temperature control ranging from 77 to 400 K and a pressure range from 10^–3^ to 10^3^ mbar. A Keithley 4200-SCS parameter analyzer was used for electrical characterization. The photoresponse was tested using a SuperK COMPACT supercontinuum white light lasers by NKT Photonics with a spectrum spanning 420–2400 nm and an optical power of up to 110 mW and using a 9072 monocromator with a bandwidth of 20 nm by Sciencetech inc.

## Supplementary Material



## Notes

Device output and transfer characteristics at various temperature, pressure and wavelength, transient photoresponse at different wavelengths, responsivity spectrum at room pressure, synaptic weight. (PDF)
