# Supplementary material for: Neuromorphic Photoresponse in Ultrathin SnS2‑Based Field Effect Transistor
Source: ACS Appl Mater Interfaces. 2025 Aug 26;17(36):50901–15. doi: 10.1021/acsami.5c11651 (PMC12442007; doi:10.1021/acsami.5c11651)
Supplement: Supplementary file 1 [file am5c11651_si_001.pdf]

## Supporting Information

# Neuromorphic photoresponse in ultrathin $SnS_2$-based field effect transistor

*Sebastiano De Stefano[1,*], Ofelia Durante[1], Andrea Sessa[1], Antonio Politano[2], Gianluca D'Olimpio[2], Tsotne Dadiani[2], Enver Faella[2], Adrian Dinescu[3], Catalin Parvulescu[3], Crispin Hetherington[4], Chia-Nung Kuo[5], Chin Shan Lue[5], Martino Aldrigo[3], Maurizio Passacantando[2], Antonio Di Bartolomeo[1,*]*

[1] Department of Physics "E. R. Caianiello", University of Salerno, Fisciano (SA), 84084, Italy

sdestefano@unisa.it, odurante@unisa.it, a.sessa96@studenti.unisa.it, adibartolomeo@unisa.it

[2] University of L'Aquila, Department of Physical and Chemical Sciences, L'Aquila, 67100, Italy

antonio.politano@univaq.it, gianluca.dolimpio@univaq.it, tsotne.dadiani@univaq.it, enver.faella@univaq.it, maurizio.passacantando@univaq.it

[3] National Institute for Research and Development in Microtechnologies, Voluntari (Ilfov), 077190, Romania

adrian.dinescu@imt.ro, catalin.parvulescu@imt.ro, martino.aldrigo@imt.ro

[4] National Center for High Resolution Electron Microscopy, Centre for Analysis and Synthesis, Lund University, Lund, SE-22100, Sweden

crispin.hetherington@chem.lu.se

[5] Department of Physics, National Cheng Kung University, Tainan 70101, Taiwan; Taiwan Consortium of Emergent Crystalline Materials (TCECM), National Science and Technology Council, Taipei 10601, Taiwan

kuochianung@gmail.com, cslue@mail.ncku.edu.tw

*Corresponding authors: sdestefano@unisa.it; adibartolomeo@unisa.it

Keywords: 2D Materials, Field Effect Transistors, Photoresponse, Trap States, Neuromorphic

**Figure S1** shows the IV characteristics shown in **Figure 2(a)** on a linear scale.

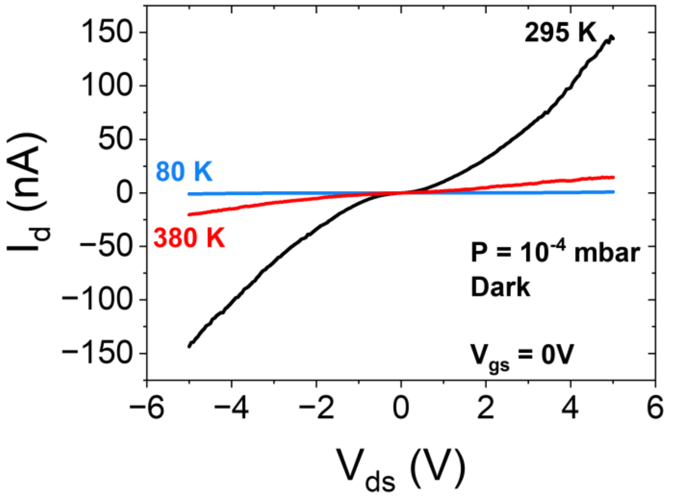


***Figure S1**: IV curves on linear scale at P = $10^{-4}$ mbar, with grounded gate and in the dark at T = 80 K (blue curve), 295 K (black curve), and 380 K (red curve).*

To elucidate the nature, donor-like or acceptor-like, of the trap states influencing the gate voltage response of the device, transfer characteristics were acquired while systematically varying the gate voltage range. **Figure S2** presents four transfer curves corresponding to $V_{gs}$ ranges incremented from ±10 V to ±40 V, in steps of 10 V. Each curve was obtained by sweeping the back-gate voltage from +40 V to –40 V and subsequently back to +40 V. The fact that the backward branch exhibits a smaller shift compared to the forward sweep as $V_{gs}$ range increases, i.e., that the hysteresis arises predominantly from a leftward shift in the threshold voltage during the forward sweep, suggests the involvement of donor-like trap states in the hysteresis mechanism [1]. This is further supported by the observed clockwise direction of the hysteresis loop.

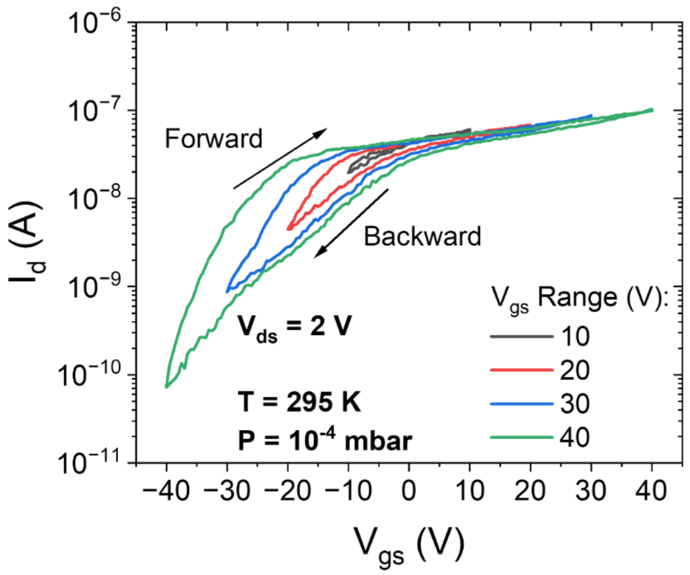


***Figure S2****: Transfer curves acquired at different gate voltage ranges; note that, as the range increases, the forward branch shifts much more to the left than the backward branch does to the right.*

**Figure S3** explains the notations for current in the main text.

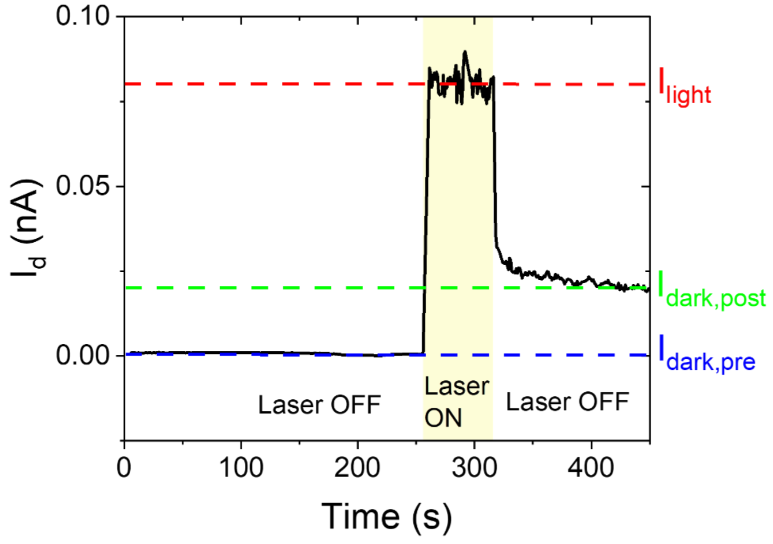


***Figure S3****: Example of $I_d$ vs. Time measurement when a laser pulse is switched on. The quantities $I_{dark,pre}$ (blue), $I_{light}$ (red), and $I_{dark,post}$ (green) are shown.*

**Figure S4** displays time-resolved current measurements (I-t) as a function of laser power at five selected excitation wavelengths (480, 520, 560, 660, and 740 nm), aimed at identifying the underlying photogeneration mechanisms. In each measurement, the laser was switched on 50 seconds after the beginning of data acquisition and remained on for 120 seconds to allow the photocurrent to reach a steady-state regime, allowing a reliable estimation of its magnitude.

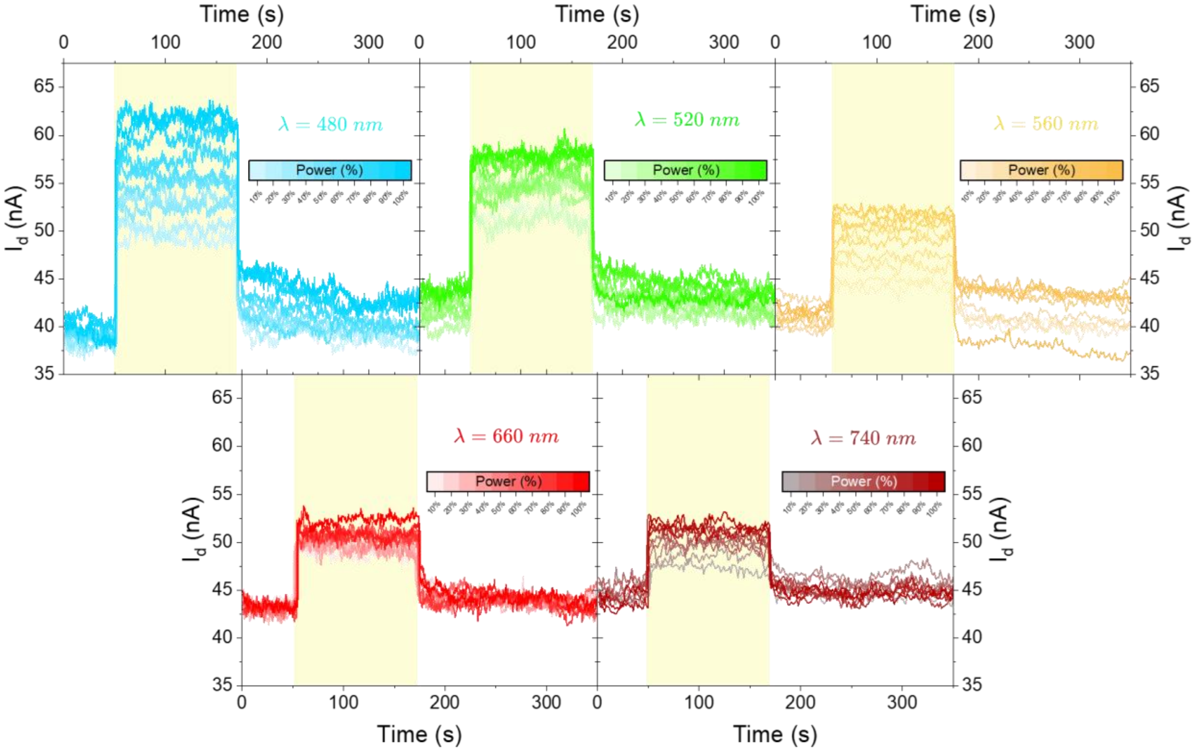


***Figure S4**: $I_d$ vs Time measurement for 480, 520, 560, 660, 740 nm incident wavelength as laser power varies, from 10% to 100% in 10% steps. They are acquired at T = 295 K and P = $10^{-4}$ mbar.*

$\log_{10}$ values of photogenerated current ($I_{ph}$) as a function of $\log_{10}$ values of incident laser power are reported in **Figure S5**, together with linear fits according to the following expression

$$log_{10}(I_{ph} - c) = A + \alpha log_{10}(P_{\lambda,inc}) \qquad (S1)$$

A progressive decrease of the slope α, along with its deviation from the ideal value of 1, is observed, indicative of the increasing influence of trap states in the photogeneration mechanisms [2,3].

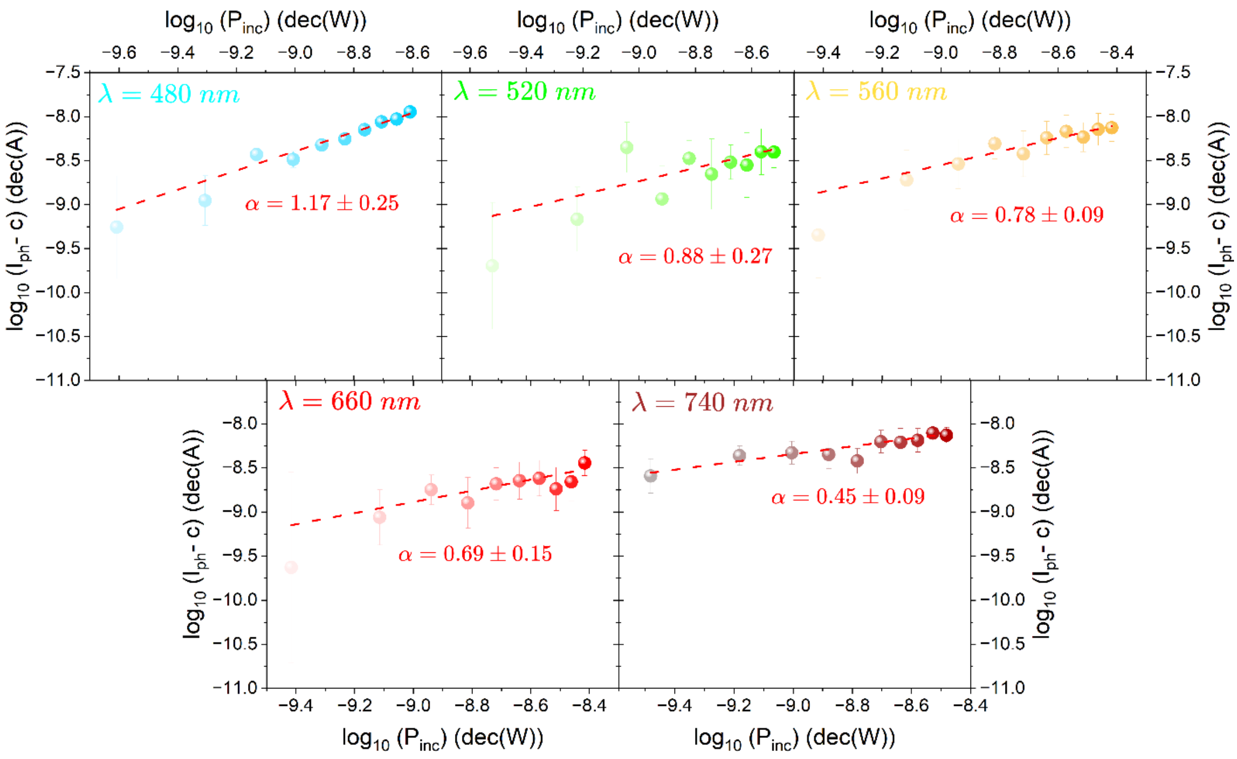


***Figure S5**: $log_{10}(I_{ph}\text{-}I_{offset})$ vs $log_{10}(P_{\lambda,inc})$ data and corresponding fit with linear fitting (red dashed line) for 480, 520, 560, 660, 740 nm. The estimated values of the α fitting parameter are also indicated.*

**Figure S6(a)** presents the transfer characteristics acquired in vacuum at T = 295 K, both in the dark and under illumination with the five previously selected wavelengths. It can be observed that, in the on state, i.e. for $V_{gs} > V_{th}$, the illuminated curves closely follow the dark curve, exhibiting only a slight increase in current due to carrier photogeneration. In contrast, for $V_{gs} < V_{th}$, the illuminated curves do not turn off as expected; instead, they remain significantly elevated and exhibit a markedly weaker dependence on the applied gate voltage. This behaviour is attributed to the photoexcitation of electrons from trap states, which results in the charging of these states and induces an additional gate effect, commonly referred to as photogating. A pronounced kink, highlighted by the red arrow in all illuminated curves, marks the transition between two regimes: one in which the effects of the applied gate and the photogating-induced gate coexist (corresponding to the on state of the transistor), and another where the influence of the applied gate is effectively screened by the photogating field. In

this latter regime, although the applied gate voltage would tend to switch the device off, the drain current exhibits a much weaker modulation with $V_{gs}$, indicating a dominant photogating effect.

**Figure S6(b)** displays two I-t measurements under 480 nm illumination, acquired at $V_{gs}$= −24 (blue curve) and $V_{gs}$= +24 V (yellow curve), with both curves aligned to the same baseline dark current level. It is evident that the current decay back to the initial dark level following the light pulse is significantly slower for −24 V, indicating the presence of gate-induced persistence.

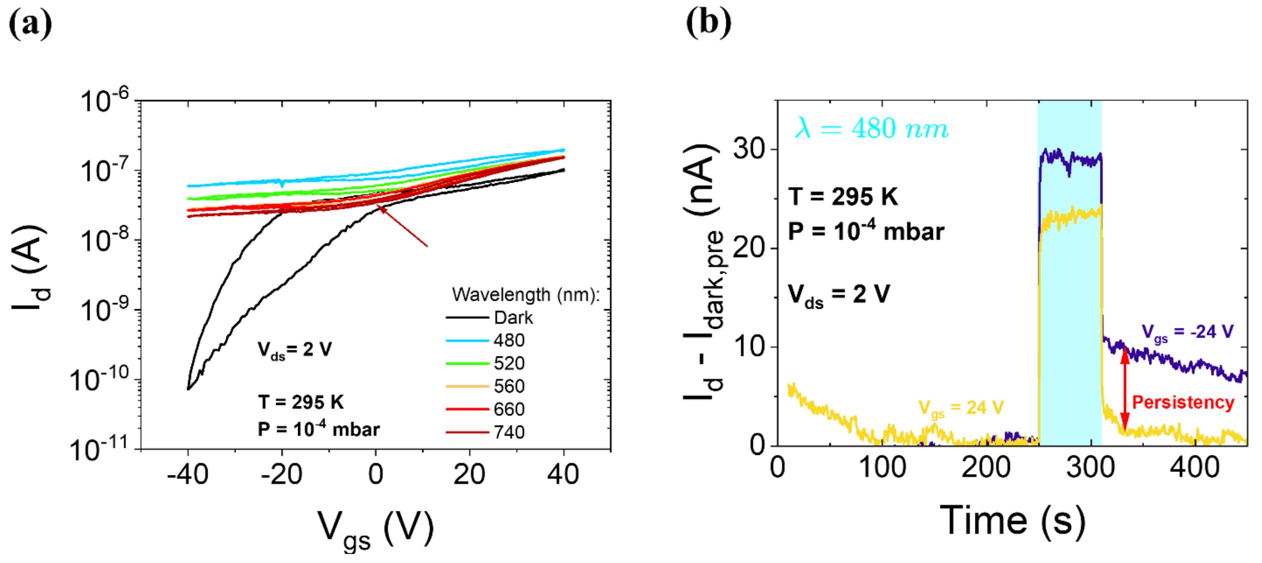


***Figure S6**: **(a)** Transfer curves acquired at T = 295 K and in vacuum, in dark (black curve) and under monochromatic light (coloured curves); **(b)** Comparison of I-t measurements at $V_{gs}$ = -24 V and +24 V placed at the same level of darkness: the red arrow highlights the persistence of current after turning off the light at -24 V compared with +24 V.*

The role of temperature in modulating the photogating effect is particularly significant. At T = 80 K, the majority of trap states are filled prior to illumination, in contrast to the situation at T = 380 K, due to thermal fluctuations. Consequently, as shown in **Figure S7**, the illumination produces a markedly larger increase in current at 80 K, as a greater number of trap states are emptied by photoexcitation. At 380 K, thermal energy enables fluctuations in the electronic energy distribution, leading to the

partial de-trapping of carriers even in the dark. As a result, fewer additional states are emptied upon illumination, yielding a reduced photogating contribution.

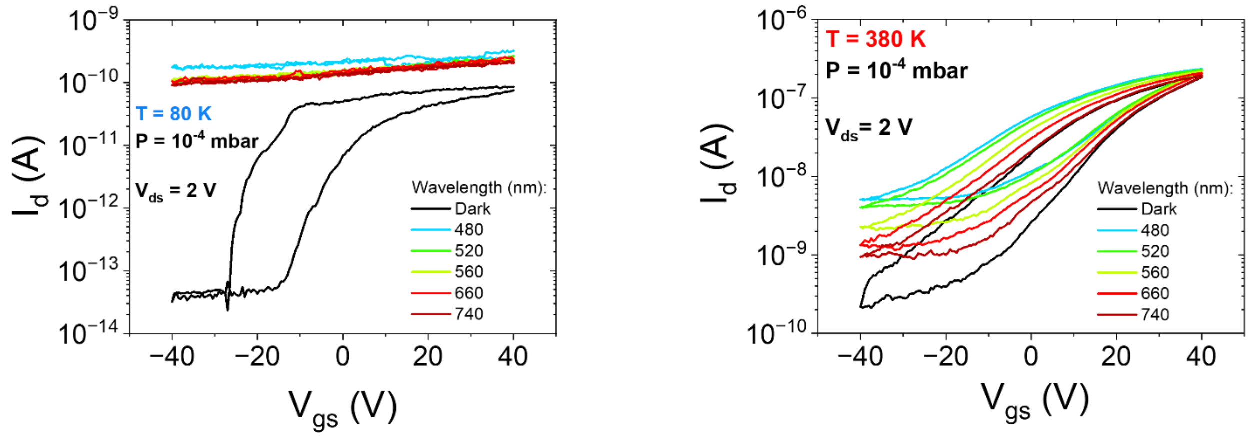


***Figure S7***: *Transfer curves acquired at T = 80 K (left) and T = 380 K (right) and in vacuum, in darkness (black curve) and under monochromatic light (coloured curves).*

**Figure S8** shows the IV characteristics shown in **Figure 7(a)** but on a linear scale.

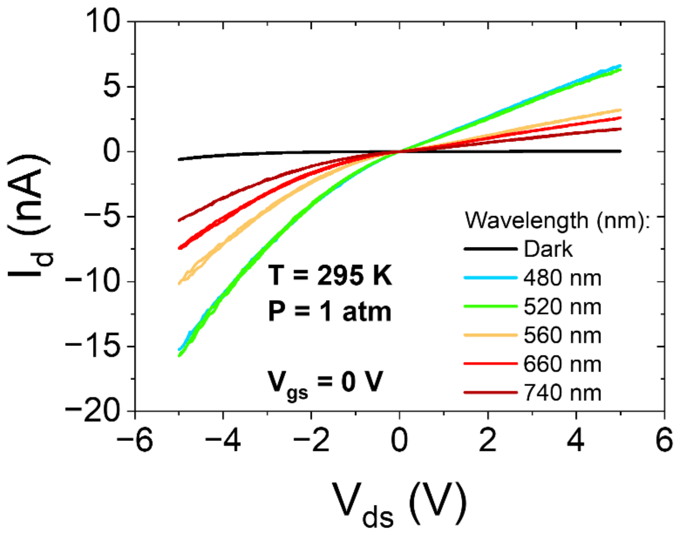


***Figure S8***: *IV characteristics on linear scale at T = 295 K and ambient pressure with grounded gate, measured both in vacuum (black curve) and under illumination (coloured curves).*

**Figure S9** reports the responsivity spectrum measured from 420 to 800 nm at T = 295 K and under ambient pressure conditions. A clear peak is observed at 480 nm, corresponding to the direct-gap transition. When compared to the spectrum shown in **Figure 3(c)**, acquired under vacuum, the measured responsivity values are noticeably lower. This reduction is likely attributable to the contribution of conduction electrons originating from adsorbed oxygen species, which results in a weaker dependence of the photocurrent on the incident optical power.

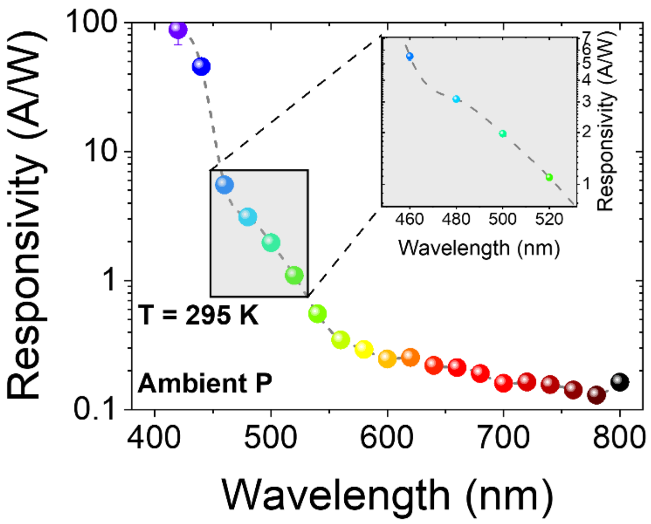


***Figure S9**: Responsivity spectrum (semi-log scale) from 420 to 800 nm, measured at T = 295 K and ambient pressure. Inset: zoom-in on the kink observed at 480 nm.*

Finally, **Figure S10** presents I-t measurements at T=295 K and ambient pressure for different gate voltages: +24 V, 0 V, and –24 V. As also observed in **Figure 4**, the applied gate voltage significantly affects the persistence of the drain current after the illumination is turned off. Specifically, more negative gate voltages result in a longer persistence of the photocurrent. Unlike the vacuum case, this

effect is also evident during the rising phase of the photocurrent: more positive gate voltages lead to a faster approach to steady-state under illumination.

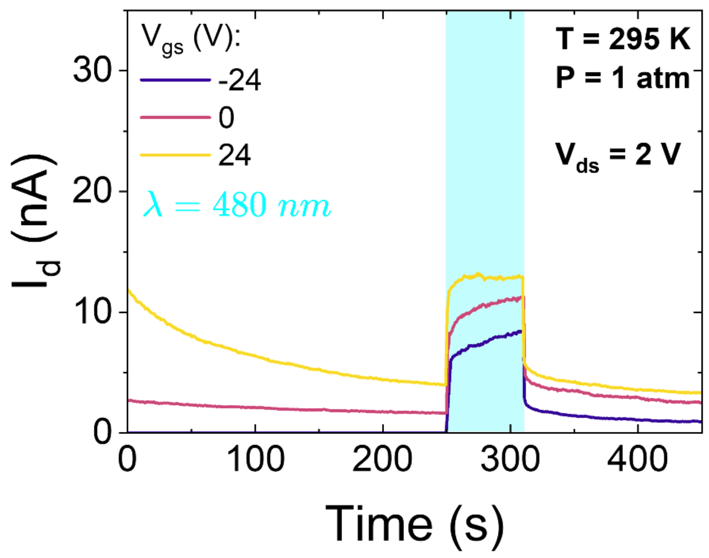


***Figure S10**: I–t measurements under 480 nm illumination at T = 295 K and ambient pressure, obtained at $V_{gs}$ = −24 V, 0 V, and 24 V.*

**Figure S11** illustrates the evolution of synaptic weights and synaptic weight changes as a function of the number of applied presynaptic pulses, corresponding to the measurements in **Figure 8(c)**. The data confirm a non-cumulative EPSC response for the selected time intervals between pulses.

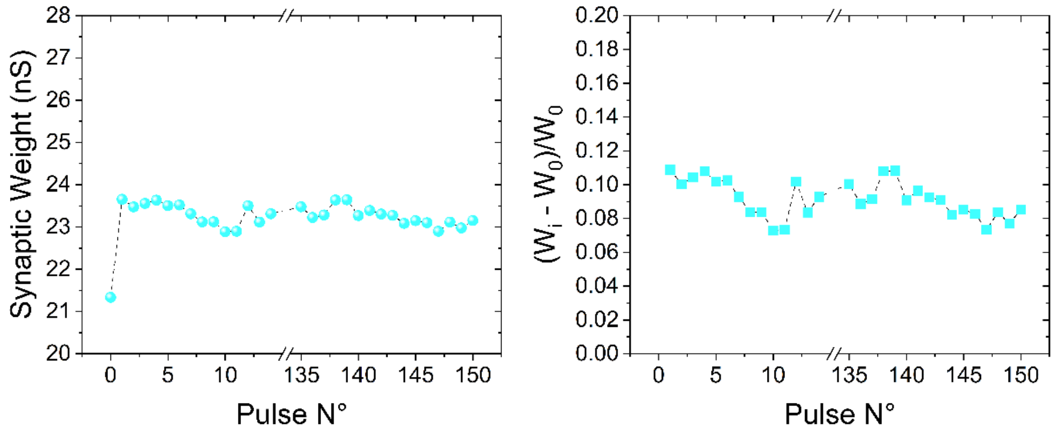


***Figure S11**: Synaptic weight (left) and Synaptic weight changes (right) evolution as a function of number of presynaptic light pulses, corresponding to the measurement of Figure 8(c).*

## Reference


1. Kim, J., Jeong, J., Lee, S., Jeong, S. & Roh, Y. Analysis of asymmetrical hysteresis phenomena observed in TMD-based field effect transistors. *AIP Adv.* **8**, 095114 (2018).
2. Ghosh, S. *et al.* Low temperature photoconductivity of few layer *p* -type tungsten diselenide ($WSe_2$ ) field-effect transistors (FETs). *Nanotechnology* **29**, 484002 (2018).
3. Buscema, M. *et al.* Photocurrent generation with two-dimensional van der Waals semiconductors. *Chem. Soc. Rev.* **44**, 3691–3718 (2015).
